# Fast myocardial T_1_ mapping using shortened inversion recovery based schemes

**DOI:** 10.1002/jmri.26649

**Published:** 2019-01-22

**Authors:** Li Huang, Radhouene Neji, Muhummad Sohaib Nazir, John Whitaker, Fiona Reid, Filippo Bosio, Amedeo Chiribiri, Reza Razavi, Sébastien Roujol

**Affiliations:** ^1^ School of Biomedical Engineering and Imaging Sciences, Faculty of Life Sciences and Medicine, King's College London United Kingdom; ^2^ MR Research Collaborations, Siemens Healthcare Limited Frimley United Kingdom; ^3^ School of Population Health and Environmental Sciences, Faculty of Life Sciences and Medicine, King's College London United Kingdom

**Keywords:** myocardial tissue characterization, T_1_ mapping, MOLLI, Look‐Locker, inversion recovery

## Abstract

**Background:**

Myocardial T_1_ mapping shows promise for assessment of cardiomyopathies. Most myocardial T_1_ mapping techniques, such as modified Look–Locker inversion recovery (MOLLI), generate one T_1_ map per breath‐held acquisition (9–17 heartbeats), which prolongs multislice protocols and may be unsuitable for patients with breath‐holding difficulties.

**Purpose:**

To develop and characterize novel shortened inversion recovery based T_1_ mapping schemes of 2–5 heartbeats.

**Study Type:**

Prospective.

**Population/Phantom:**

Numerical simulations, agarose/NiCl_2_ phantom, 16 healthy volunteers, and 24 patients.

**Field Strength/Sequence:**

1.5T/MOLLI.

**Assessment:**

All shortened T_1_ mapping schemes were characterized and compared with a conventional MOLLI scheme (5‐(3)‐3) in terms of accuracy, precision, spatial variability, and repeatability.

**Statistical Tests:**

Kruskal–Wallis, Wilcoxon rank sum tests, analysis of variance, Student's *t*‐tests, Bland–Altman analysis, and Pearson correlation analysis.

**Results:**

All shortened schemes provided limited T_1_ time variations (≤2% for T_1_ times ≤1200 msec) and limited penalty of precision (by a factor of ~1.4–1.5) when compared with MOLLI in numerical simulations. In phantom, differences between all schemes in terms of accuracy, spatial variability, and repeatability did not reach statistical significance (*P* > 0.71). In healthy volunteers, there were no statistically significant differences between all schemes in terms of native T_1_ times and repeatability for myocardium (*P* = 0.21 and *P* = 0.87, respectively) and blood (*P* = 0.79 and *P* = 0.41, respectively). All shortened schemes led to a limited increase of spatial variability for native myocardial T_1_ mapping with respect to MOLLI (by a factor of 1.2) (*P* < 0.0001). In both healthy volunteers and patients, the two‐heartbeat scheme and MOLLI led to highly linearly correlated T_1_ times (correlation coefficients ≥0.83).

**Data Conclusion:**

The proposed two‐heartbeat T_1_ mapping scheme yields a 5‐fold acceleration compared with MOLLI, with highly linearly correlated T_1_ times, no significant difference of repeatability, and limited spatial variability penalty at 1.5T. This approach may enable myocardial T_1_ mapping in patients with severe breath‐holding difficulties and reduce the examination time of multislice protocols.

**Level of Evidence**: 1

**Technical Efficacy Stage**: 3

J. Magn. Reson. Imaging 2019;50:641–654.

Native myocardial longitudinal relaxation time (T_1_) is sensitive to a wide range of cardiomyopathies.^1^ This biomarker is commonly estimated on a per‐voxel basis, which is referred to as myocardial T_1_ mapping.^2^ Myocardial T_1_ maps can also be performed before and after administration of a gadolinium‐based contrast agent. The combination of native and postcontrast myocardial and blood T_1_ times enables the estimation of the extracellular volume (ECV) fraction,^3^ which has important diagnostic and prognostic value.^4^

A variety of imaging sequences have been proposed for myocardial T_1_ mapping and often use magnetization preparation pulses such as inversion,^2^, ^5^, ^6^ saturation,^7^, ^8^, ^9^, ^10^, ^11^ or hybrid pulses.^12^, ^13^ In these techniques, a series of images with different T_1_‐weightings is acquired and followed by voxel‐wise fitting to a model of the measured signal to generate a T_1_ map.^2^ The modified Look–Locker inversion recovery (MOLLI) sequence^2^ and its variations, such as the shortened MOLLI (ShMOLLI)^5^ and other modified MOLLI schemes,^16^ are inversion recovery based techniques. Although MOLLI T_1_ times have been shown to be dependent on several parameters including T_2_,^14^, ^18^ magnetization transfer,^19^ off‐resonance,^16^ inversion factor,^20^ and heart rate (HR),^16^ this approach is commonly used for myocardial T_1_ mapping due to its high reproducibility/high repeatability, high precision/low spatial variability, and high map quality/low artifact level.^14^, ^15^, ^16^, ^17^

Typical MOLLI sequences consist of several inversion pulses, each followed by a series of electrocardiogram (ECG)‐triggered single‐shot acquisitions. A variety of MOLLI schemes have been proposed using different amounts of T_1_‐weighted images and inversion pulses.^2^, ^5^, ^16^ T_1_ map reconstruction of MOLLI sequences commonly uses a three‐parameter (3P) fitting model of the inversion recovery signal^2^ followed by a Look–Locker correction.^21^ During the fitting process, the signal polarity can be restored using a multifitting approach^2^ or a phase‐sensitive inversion recovery (PSIR) reconstruction.^22^ Alternative MOLLI reconstructions have been proposed using more complex models^23^ or Bloch equations simulation of the sequences.^24^, ^25^, ^26^

The common acquisition manner in MOLLI sequences is a single T_1_ map per breath‐hold, thus limiting the total measurement time. Most MOLLI schemes acquire data over 9–17 heartbeats.^2^, ^5^, ^16^ However, breath‐holding capabilities may be as low as 2 seconds in patients with cardiac or respiratory disease.^27^ Therefore, shortened breath‐held acquisition may be beneficial to such patients. Furthermore, the required spatial coverage of myocardial T_1_ mapping (from single slice to full ventricular coverage) may also depend on the pathology being assessed.^1^ MOLLI T_1_ mapping with full ventricular coverage requires repeated breath‐held acquisitions, each for a single slice, thus increasing patient discomfort and prolonging scan time. Therefore, shorter breath‐holding requirement for myocardial T_1_ mapping would be advantageous for multislice T_1_ mapping protocols.

In this work, we sought to develop and characterize novel shortened inversion recovery based T_1_ mapping schemes of 2–5 heartbeats.

## Materials and Methods

All imaging was performed using a 1.5T MR scanner (Magnetom Aera, Siemens Healthcare, Erlangen, Germany). This work was conducted according to the Declaration of Helsinki and Good Clinical Practice guidelines and was approved by a local Research Ethics Committee (approval number 01/11/12 for the healthy volunteer study and 15/NS/0030 for the patient study). Informed consent was obtained from all participants.

### *T_1_ Mapping Schemes*

Several shortened T_1_ mapping schemes were evaluated using two, three, four, or five ECG‐triggered single‐shot images following a single inversion pulse (see Supplementary Material 1). Both magnitude and phase images were reconstructed from these acquisitions. A short inversion time (TI) of TI_min_ = 100 msec was used for the first image.

Two T_1_ fitting reconstructions using a novel two‐parameter (2P) fitting model and a standard 3P fitting model were evaluated. 2P‐n (*n* = 2–5) and 3P‐n (*n* = 3–5), hereafter referred to as T_1_ mapping using *n* images following a single inversion pulse with the 2P and 3P fitting models, respectively. These schemes were compared with a conventional 5‐(3)‐3 MOLLI scheme (i.e., 3P‐8). Note that 3P‐5 can be seen as an approximation of ShMOLLI for native myocardial T_1_ mapping.^16^

### *T_1_ Map Reconstruction*

#### *PROPOSED 2P FITTING MODEL*

For T_1_ fitting, an exhaustive search was performed over a normalized signal dictionary created using the proposed following model:

(1) $$S_{\text{dict}}\left(\mathrm{TI}\right)=1-\left(1+\delta \right)\cdot e^{-\mathrm{TI}/T_{1}},$$

where δ ≤ 1 is a constant term representing the inversion factor of the inversion pulse. δ was determined by Bloch equations simulation of the employed nonselective tuned inversion pulse (phase‐modulated hyperbolic tangent, duration 2.56 msec, frequency sweep 9.5 KHz, ζ = 10, tanκ = 22 with a flip angle of 300°, i.e., a peak B_1_ strength of 14.4 μT) over typical native and postcontrast myocardial T_1_ ranges ([400,1600] msec), B_0_ field inhomogeneity ([–150,+150] Hz), B_1_ field inhomogeneity ([80%,100%]) and a typical myocardial T_2_ time of 45 msec.^20^ The effective flip angle was approximated based on the average longitudinal magnetization over the slice profile and all simulated T_1_/T_2_/B_0_/B_1_ regimes. The average inversion factor δ was estimated as 0.9633. The signal dictionary S_dict_(TI) was created for a 1‐msec‐step T_1_ range of [100,2200] msec, which covers the entire range of native and postcontrast myocardial and blood T_1_ times.

Before dictionary matching, the polarity‐restored measured signal S_restored_(TI_j_) (j = 1,2,…8) was computed from the measured signal S_meas_(TI_j_) (j = 1,2,…8) using a modified PSIR approach. The first image with the shortest TI (TI_1_ = TI_min_ = 100 msec), which is one of the only two common images among all schemes, was chosen as the reference phase image with "negative" polarity (i.e., S_restored_(TI_1_) = –S_meas_(TI_1_)). Using Bloch equations simulation of the sequence, this assumption was valid for any T_1_ time > 172 msec (in the presence of any T_2_ times ≥30 msec and imaging flip angles ≤85°), thus including the entire physiological ranges of native/postcontrast T_1_ times in myocardium, blood, and fat (see Supplementary Material 2 for more details). As the signal dictionary is normalized, the polarity‐restored measured signal was individually scaled $\left(S_{\text{restored}}^{\left(\text{scaled}\right)}\left({\mathrm{TI}}_{j}\right)\right)$ to each dictionary entry S_dict_(TI_j_) as:

(2) $$S_{\text{restored}}^{\left(\text{scaled}\right)}\left({\mathrm{TI}}_{j}\right)=S_{\text{restored}}\left({\mathrm{TI}}_{j}\right)\cdot \frac{\bar{\left|S_{\text{dict}}\right|}}{\bar{\left|S_{\text{restored}}\right|}}=S_{\text{restored}}\left({\mathrm{TI}}_{j}\right)\cdot \frac{\sum_{j=1}^{n}\left|S_{\text{dict}}\left({\mathrm{TI}}_{j}\right)\right|}{\sum_{j=1}^{n}\left|S_{\text{restored}}\left({\mathrm{TI}}_{j}\right)\right|},$$

where n is the amount of T_1_‐weighted images in the 2P‐n scheme, $\bar{\left|S_{\text{dict}}\right|}$ and $\bar{\left|S_{\text{restored}}\right|}$ are the signal amplitude averages over all TIs (TI_1_‐TI_n_) of a dictionary entry and the polarity‐restored measured signal, respectively. Dictionary matching was finally performed by minimizing the L2‐norm between $S_{\text{restored}}^{\left(\text{scaled}\right)}$ and each dictionary entry.

This reconstruction was implemented on an affordable graphics processing unit (GPU) (NVIDIA, Quadro K620 2GB) using the compute unified device architecture (CUDA) to enable high‐performance computing. The parallelization level was set to the pixel level. This implementation was compared with a central processing unit (CPU)‐only implementation entirely developed in C++. The resulting T_1_ times were subsequently HR‐corrected as described in the second next section.

#### *3P FITTING MODEL*

The signal of the 3P fitting model is defined as:

(3) $$S\left(\mathrm{TI}\right)=A-B\cdot e^{-\mathrm{TI}/T_{1}^{*}},$$

where A, B, and $T_{1}^{*}$ are the model parameters.^2^ Note that $T_{1}^{*}$ is often referred to as the apparent T_1_ time. A PSIR reconstruction was employed to restore the signal polarity as described above. A Levenberg–Marquardt solver, provided previously,^28^ was used for simultaneous estimation of A, B, and $T_{1}^{*}$. T_1_ times were then approximated as:

(4) $$T_{1}=T_{1}^{*}\cdot \left(\frac{B}{A}-1\right),$$

as proposed previously.^2^ Finally, a correction for imperfect inversion was performed by dividing T_1_ times with δ, the inversion factor of the inversion pulse as described above.^20^ A subsequent HR correction on the resulting T_1_ times was performed as described in the next section.

### *Heart Rate Correction*

Myocardial T_1_ times using MOLLI have been shown to be HR‐dependent.^16^, ^29^, ^30^ In this work, a novel approach for correction of HR‐dependent T_1_ errors is proposed for each of the eight evaluated T_1_ mapping scheme 2P‐n (*n* = 2–5) and 3P‐n (*n* = 3–5) as well as MOLLI. This correction approach was created using phantom experiments in nine agarose/NiCl_2_ vials with different T_1_/T_2_ times representing typical T_1_/T_2_ ranges of native and postcontrast myocardium and blood (T1MES, Resonance Health, Burswood, WA, Australia). Imaging parameters are described in the next section. The T_1_ dependence on HR of each T_1_ mapping scheme is shown in Supplementary Material 3 for each vial. Different linear dependence of measured T_1_ over physiological HR (40–120 bpm) was observed for different T_1_ mapping schemes, which is vial‐ and thus T_1_‐dependent. Individual linear regressions were performed for each T_1_ mapping scheme and each vial, leading to different slopes and offsets for each T_1_ mapping scheme and vial (see Supplementary Material 3), as described below:

(5) $$T_{1}=\text{slope}\left(T_{1}\right)\cdot \mathrm{HR}+\text{offset}\left(T_{1}\right).$$

Note that this relationship is also T_2_‐dependent. Therefore, two different correction models were developed for myocardium (using short‐T_2_ vials with T_2_~45 msec) and blood (using long‐T_2_ vials with T_2_ > 150 msec), respectively. Each correction model was created as follows. An empirical method was established to correct the HR‐dependent T_1_ errors by aligning measured T_1_ times to the value at a theoretical HR of 60 bpm (T_1_^(corr)^) based on:

(6) $$\frac{T_{1}-\text{offset}\left(T_{1}\right)}{\mathrm{HR}}=\frac{T_{1}-{T_{1}}^{\left(\text{corr}\right)}}{\mathrm{HR}-60}=\text{slope}\left(T_{1}\right).$$

To make this model applicable to any T_1_ times (and not limited to the ones corresponding to the phantom), a parabolic relationship between slope, offset, and T_1_ was empirically defined as:

(7) $$\text{slope}\left(T_{1}\right)=a_{1}\cdot {\left[\text{offset}\left(T_{1}\right)\right]}^{2}+a_{2}\cdot \text{offset}\left(T_{1}\right)+a_{3},$$

where a_1_, a_2_, and a_3_ are the coefficients of the parabolic function and were obtained from least square fitting (see Supplementary Material 4). Using Eq. (6), Eq. (7) can then be rewritten as:

(8) $$a_{1}\cdot {\left[\text{offset}\left(T_{1}\right)\right]}^{2}+\left(a_{2}+\frac{1}{\mathrm{HR}}\right)\cdot \text{offset}\left(T_{1}\right)+\left(a_{3}-\frac{T_{1}}{\mathrm{HR}}\right)=0.$$

In such case, the offset can be derived as:

(9) $$\text{offset}\left(T_{1}\right)=\frac{1}{2a_{1}}\left[-\left(a_{2}+\frac{1}{\mathrm{HR}}\right)\pm \sqrt{{\left(a_{2}+\frac{1}{\mathrm{HR}}\right)}^{2}-4\cdot a_{1}\cdot \left(a_{3}-\frac{T_{1}}{\mathrm{HR}}\right)}\right],$$

where the positive root (" + " instead of " ± ") was found to provide a physiologically reasonable offset. Then the corrected T_1_ (T_1_^(corr)^) can be computed from Eqs. 6, 9 as:

(10) $${T_{1}}^{\left(\text{corr}\right)}=T_{1}-\frac{\mathrm{HR}-60}{\mathrm{HR}}\cdot \left[T_{1}-\text{offset}\left(T_{1}\right)\right].$$

### *Experimental Validation*

#### *Numerical Simulations*

Numerical simulations were used to study the T_1_ accuracy and precision of the proposed shortened T_1_ mapping schemes and the conventional 5‐(3)‐3 MOLLI scheme. The Bloch equations were used to simulate the signal of each sequence by measuring the simulated transverse magnetization at the *k*‐space center of each imaging readout. All numerical simulations used a simulated HR of 60 bpm and the following imaging parameters: TR/TE/TI_1_/TI_2_ = 2.7/1.1/100/180 msec, 62 phase encoding lines in linear ordering, partial Fourier factor = 7/8, and five start‐up pulses. The slice profile of the employed excitation pulse (Hann‐filtered sinc pulse without phase modulation, duration 0.48 msec, bandwidth 4660 Hz, time‐bandwidth product 1.6, prescribed flip angle 35°, peak strength 10.9 μT) was estimated by Bloch equations simulation over the same T_1_/T_2_ ranges and B_0_/B_1_ inhomogeneities as used for the inversion pulse. A resulting average excitation flip angle of 26° was obtained and used for the simulations. An average inversion flip angle of 164° corresponding to δ = 0.9633 was also used for the simulations.

Numerical simulations were performed over a range of typical myocardial T_1_ times (300–1500 msec in steps of 50 msec) and myocardial T_2_ times (30–70 msec in steps of 5 msec). Monte‐Carlo simulation (*N* = 50,000) were performed for each pair of simulated T_1_/T_2_ times using random noise corresponding to a signal‐to‐noise ratio (SNR) of 50 in the longest‐TI image of the conventional MOLLI scheme (TI_max_ = 4100 msec). Accuracy was assessed as the average over the *N* repetitions of the difference between the simulated and estimated T_1_ times. Precision was defined as the standard deviation (SD) of the estimated T_1_ times over the *N* repetitions.

To evaluate the influence of SNR on T_1_ accuracy and precision, additional numerical simulations were performed for different SNR ([10,25,50,100]) and T_1_ range (300–1500 msec in steps of 50 msec), and a fixed T_2_ times of 45 msec.

#### *PHANTOM STUDY*

The proposed 2P fitting model was characterized and compared with the conventional 3P fitting model using different shortened T_1_ mapping schemes and the conventional 5‐(3)‐3 MOLLI scheme in a phantom with nine agarose/NiCl_2_ vials of different T_1_/T_2_ times in the ranges for native and postcontrast myocardium and blood (T1MES, Resonance Health). To this end, the conventional 5‐(3)‐3 MOLLI acquisition scheme was used. The first two to five ECG‐triggered single‐shot images following the first inversion pulse were used for 2P‐n (*n* = 2–5) and 3P‐n (*n* = 3–5). The conventional MOLLI reconstruction using all images (i.e., 3P‐8) was also performed. The 2D balanced steady‐state free precession (bSSFP) imaging readout used the following parameters: TR/TE/flip angle = 2.7 msec/1.1 msec/35°, field of view (FOV) = 360 × 306 mm^2^, voxel size = 1.4 × 2.1 mm^2^, three slices, slice gap = 8 mm, slice thickness = 8 mm, GRAPPA factor = 2, partial Fourier factor = 7/8, bandwidth = 1085 Hz/px, 62 phase‐encoding lines in linear ordering, and five start‐up pulses.

Experiment #1: Characterization of T_1_ accuracy, spatial variability, and repeatability. The 5‐(3)‐3 MOLLI acquisition scheme with a simulated HR of 60 bpm was repeated five times for assessment of T_1_ accuracy, spatial variability, and repeatability of all schemes. The reference T_2_ times were obtained from the manufacturer. The reference T_1_ times were obtained using inversion recovery based spin echo T_1_ mapping (TI = [50, 100, 200, 300, 400, 500, 600, 700, 800, 900, 1000, 2000, 3000, 4000, 5000] msec, TE/TR = 15/15000 msec). A region of interest (ROI) was manually drawn for each vial. Measured T_1_ times were obtained for each vial as the averages over the five repetitions of the mean T_1_ times in the corresponding ROI. T_1_ accuracy was measured as the difference between measured and reference T_1_ times. T_1_ spatial variability was measured for each vial as the average over the five repetitions of the SD of T_1_ times in the corresponding ROI. T_1_ repeatability was estimated for each vial as the SD over the five repetitions of the mean T_1_ times in the corresponding ROI.

Experiment #2: Characterization of the proposed HR correction. The performance of the proposed HR correction was evaluated using a second dataset of measurements where the 5‐(3)‐3 MOLLI scheme was acquired with different simulated HRs ([40–120] bpm in steps of 10 bpm). All 2P‐n and 3P‐n reconstructions were performed without and with HR correction using the two different correction models for short‐T_2_ and long‐T_2_ vials. Note that the data used for creating the HR correction models were obtained from a separated study performed earlier on a different day. T_1_ variation as the average absolute differences with respect to the value at the reference HR of 60 bpm, described as:

(11) $$\text{mean}\left\{\left|\Delta_{\mathrm{HR}}T_{1}\right|\right\}=\bar{\left|T_{1}\left(\mathrm{HR}\right)-T_{1}\left(60\right)\right|},$$

was calculated for pre‐ and post‐HR‐correction on each vial, in order to indicate the T_1_ mapping sensitivity to HR of each evaluated T_1_ mapping schemes.

#### *HEALTHY VOLUNTEER STUDY*

In vivo characterization was performed in 16 healthy volunteers (seven male, 28 ± 3 years). Native myocardial T_1_ mapping was performed using the 5‐(3)‐3 MOLLI acquisition scheme and the imaging parameters described in the phantom study. This protocol was modified to acquire three slices in the short axis orientation, each in a separated breath‐hold. This acquisition was repeated twice for each healthy volunteer. All 2P‐n and 3P‐n reconstructions were performed without and with HR correction using the short T_2_ and long T_2_‐based correction models for myocardial and blood T_1_ analyses, respectively.

Myocardial T_1_ analysis was based on a 16‐myocardial‐segment model,^31^ while blood T_1_ analysis was based on a single ROI drawn inside the left ventricular blood pool in the basal slice with careful exclusion of the papillary muscles. A representative example of ROIs used for myocardial and blood T_1_ quantification is shown in Supplementary Material 5. All data were visually inspected to detect the presence of severe artifacts or motion among the T_1_‐weighted images. Myocardial segments with apparent severe artifacts in the MOLLI T_1_ maps were discarded from quantitative myocardial T_1_ analysis of all schemes. Myocardial and blood T_1_ times, spatial variability, and repeatability were assessed for each subject. A segment‐wise T_1_ time was calculated as the average over the two repetitions of the T_1_ mean in each myocardial segment and blood pool. Segment‐wise T_1_ spatial variability was measured as the average over the two repetitions of the T_1_ spatial SD in each myocardial segment and blood pool. Segment‐wise T_1_ repeatability was estimated as the absolute difference between the two repetitions of the T_1_ mean in each myocardial segment and blood pool. The corresponding subject‐wise T_1_ time, spatial variability, and repeatability were computed as the averages over all nondiscarded segments, respectively.

#### *Patient Study*

Twenty‐four consecutive patients (17 male, 53 ± 17 years) referred for clinical cardiac MRI in our center were recruited. Native myocardial T_1_ mapping was performed in all patients. Eighteen of these patients (13 male, 53 ± 19 years) received an injection of 0.1 mmol/kg of gadobutrol (Gadovist, Bayer Vital, Leverkusen, Germany) as part of the clinical protocols. Postcontrast T_1_ mapping was thus also performed in these patients. Native and postcontrast myocardial T_1_ mapping were performed using the same 5‐(3)‐3 MOLLI acquisition scheme and imaging parameters described in the healthy volunteer study. Three slices were acquired in the short axis orientation, each in a separated breath‐hold. All 2P‐n and 3P‐n reconstructions were performed without and with HR correction using the short T_2_ and long T_2_‐based correction models for myocardial and blood T_1_ analyses, respectively. Subject‐wise myocardial and blood T_1_ times were measured as described in the healthy volunteers section.

### *Statistical Analysis*

Kruskal–Wallis test and a one‐way analysis of variance (ANOVA) test were used to compare all T_1_ mapping schemes in phantom and in vivo, respectively. A result was considered statistically significant at the 5% significance level (i.e., *P* < 0.05) and all tests were two‐tailed. When the Kruskal–Wallis or one‐way ANOVA test demonstrated statistical significance, Wilcoxon rank sum tests or Student's *t*‐tests were performed for each pair of T_1_ mapping schemes using Bonferroni correction, which resulted in a statistical significance threshold of 0.05/C^2^_8_ ≈ 0.0018. Correlation and agreement analyses in the form of Pearson correlation analysis and Bland–Altman plots with limits of agreement, respectively, were performed between each shortened T_1_ mapping scheme and MOLLI in terms of subject‐wise native/postcontrast myocardial/blood T_1_ times. Bland–Altman limits of agreement were calculated as the mean difference between methods (also called bias) ± 1.96 × (SD of differences); ~95% of differences between methods should lie within these limits.

## Results

### *Computational cost of the 2P model‐based reconstruction*

2P‐2, 2P‐3, 2P‐4, and 2P‐5 reconstruction times for one T_1_ map (256 × 256 matrix size) were 7, 8, 11, and 13 seconds using a CPU‐based implementation, respectively. These reconstruction times were reduced to 0.2 seconds for all 2P reconstructions using the proposed GPU‐based implementation. Reconstruction times for all CPU‐based implementations increased linearly with the number of slices, while GPU‐based reconstruction times increased at a slower rate. For example, 2P‐2, 2P‐3, 2P‐4, and 2P‐5 reconstruction times for 10 T_1_ maps (256 × 256 × 10 matrix size) were 65, 83, 104, and 130 seconds using the CPU‐based implementation, and were reduced to 1, 1.3, 1.7, and 2 seconds using the proposed GPU‐based implementation, respectively.

### *Numerical Simulations*

**Figure 1:**
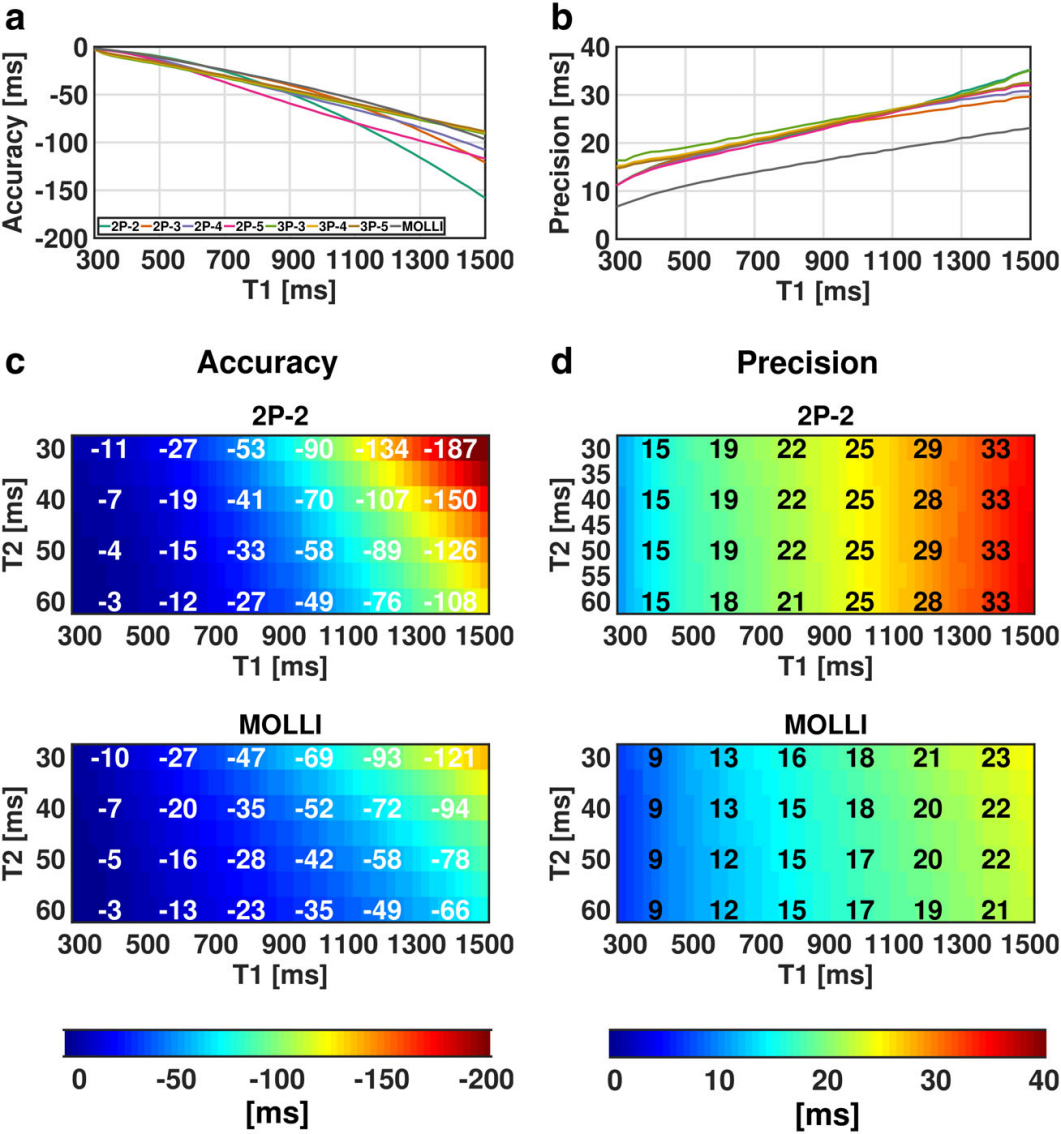
Numerical simulations of T_1_ accuracy and precision of all T_1_ mapping schemes. T_1_ accuracy **(a)** and precision **(b)** are shown as a function of T_1_ using a typical myocardial T_2_ time of 45 msec for all T_1_ mapping schemes. Impact of T_2_ times on T_1_ accuracy **(c)** and precision **(d)** are shown for 2P‐2 and MOLLI.

Accuracy and precision of all evaluated T_1_ mapping schemes are shown in Fig. 1. All 3P‐n schemes led to limited T_1_ time variation (≤2%) with respect to MOLLI for the entire range of T_1_ times ([300,1500] msec). All 2P‐n schemes provided limited T_1_ time variations (≤2%) with respect to MOLLI for T_1_ times ≤1200 msec but resulted in reduced accuracy for longer T_1_ times. All shortened T_1_ mapping schemes led to a precision penalty with respect to MOLLI by a factor of ~1.4–1.5. All studied schemes were T_2_‐dependent. Lower T_2_ times were associated with decreased accuracy for all schemes.

SNR had limited influence on the T_1_ time estimates of all schemes (variation ≤2% with respect to T_1_ estimations with an SNR of 100, see Supplementary Material 6). Lower SNR resulted in a reduced T_1_ precision of all schemes. However, SNR had limited influence on the relative precision penalty of all shortened T_1_ mapping schemes with respect to MOLLI, which was by a factor of 1.4–1.5 for the entire SNR range.

### *Phantom Study*

**Figure 2:**
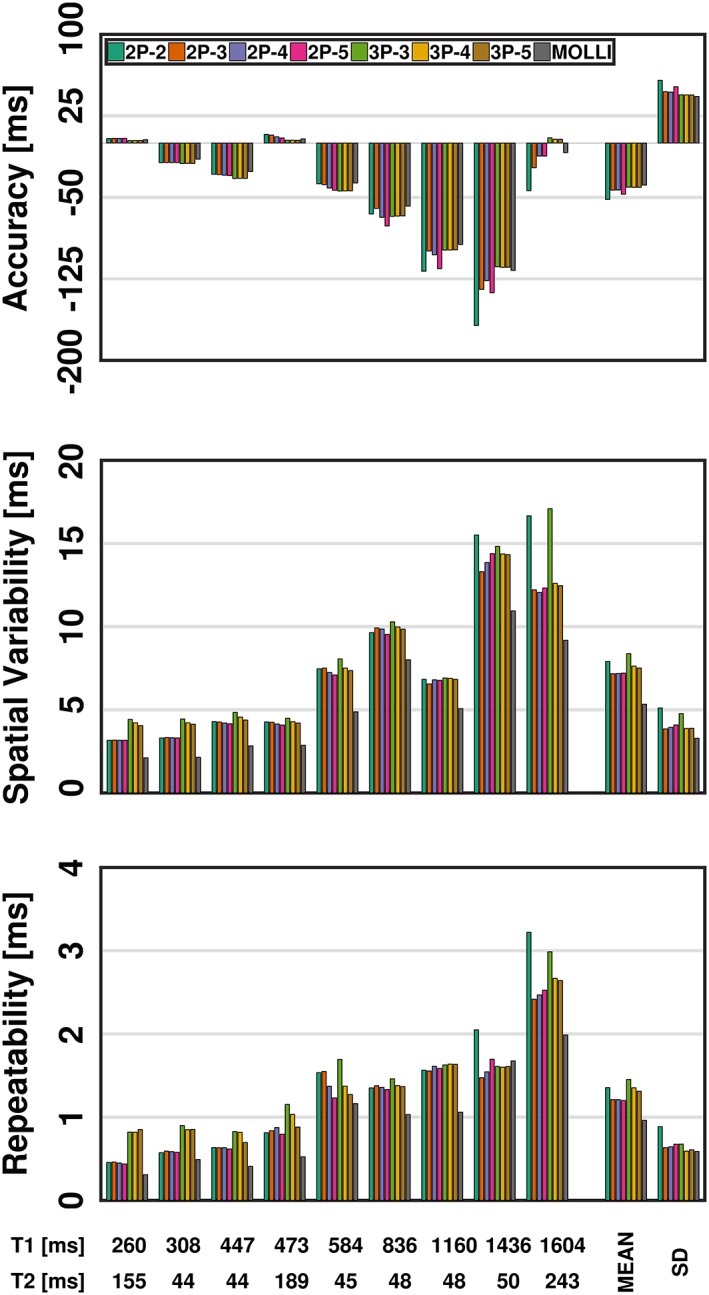
T_1_ accuracy **(a)**, spatial variability **(b)**, and repeatability **(c)** of all T_1_ mapping schemes in phantom experiments. There were no statistically significant differences between all schemes in terms of accuracy (*P* = 1.00), spatial variability (*P* = 0.71) and repeatability (*P* = 0.75).

Experiment #1: Characterization of T_1_ accuracy, spatial variability, and repeatability. T_1_ accuracy, spatial variability, and repeatability in phantom using all evaluated T_1_ mapping schemes (conventional MOLLI and shortened T_1_ mapping schemes: 2P‐n [*n* = 2–5] and 3P‐n [*n* = 3–5]) are shown in Fig. 2. All schemes were in good agreement with the reference T_1_ times for long‐T_2_ vials (i.e., T_2_ > 150 msec) with an average error of <11 msec for all schemes. All schemes led to underestimated T_1_ times for short‐T_2_ vials (i.e., T_2_~45 msec) with respect to the reference T_1_ times. Although MOLLI tended to provide slightly lower underestimation than shortened T_1_ mapping schemes (especially for short‐T_2_ vials), these differences were not statistically significant (*P* = 1.00). For a typical native myocardial T_1_ range (the vial with T_1_/T_2_ 1160/48 msec), 2P‐2 and 2P‐5 led to an underestimation of 25 msec and 22 msec with respect to MOLLI, while the other shortened T_1_ mapping schemes led to an underestimation of <10 msec. The 2P‐n (*n* = 2–5) schemes tended to provide lower spatial variability than the 3P‐n (*n* = 3–5) schemes for typical postcontrast T_1_ range (<450 msec), while 2P‐2 and 3P‐3 tended to show higher spatial variability than other schemes for long T_1_ times (>1400 msec). Although all schemes tended to provide higher spatial variability than MOLLI (7–8 msec vs. 5 msec, respectively), and lower repeatability than MOLLI (1.2–1.5 msec vs. 1.0 msec, respectively), these differences were not statistically significant (*P* = 0.71 and *P* = 0.75, respectively).

Experiment #2: Characterization of the proposed HR correction. Supplementary Material 7 shows the impact of the proposed HR correction for measured T_1_ times. After the proposed HR correction, T_1_ variation over all HR was reduced from a maximum of 55 msec to a maximum of 7 msec for all vials and T_1_ mapping schemes.

### *Healthy Volunteer Study*

**Figure 3:**
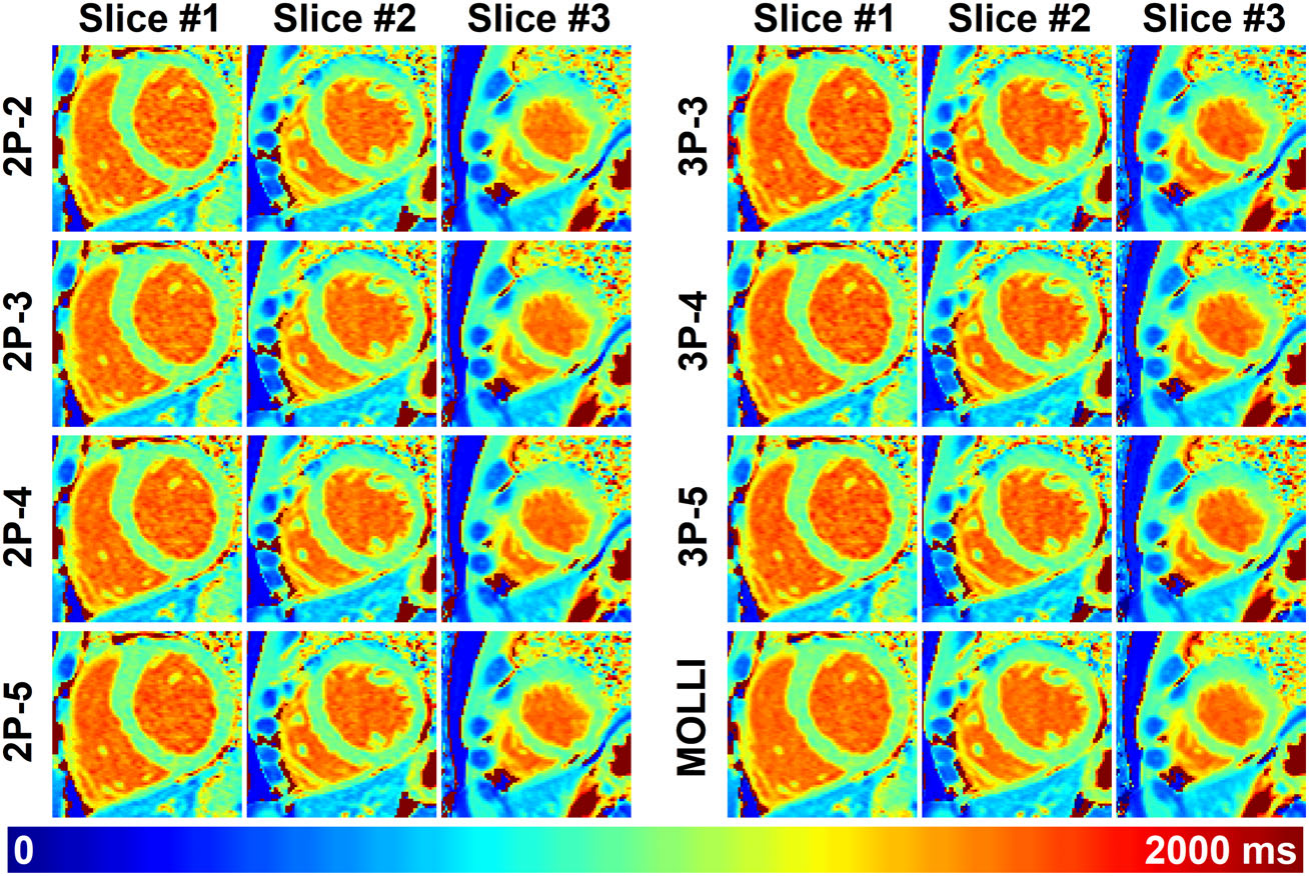
Example native T_1_ maps of a 29‐year‐old male healthy volunteer (HR 52 bpm) along the short axis using all T_1_ mapping schemes. Similar image quality and native T_1_ range were obtained across all slices using all schemes.

Example native myocardial T_1_ maps of a healthy volunteer using all T_1_ mapping schemes are shown in Fig. 3. All schemes provided similar visual image quality across all slices and segments, as well as similar native T_1_ ranges for myocardium and blood. The perceived noise, however, was higher in the left ventricular blood pool for all shortened T_1_ mapping schemes.

The average HR over all healthy volunteers was 68 ± 12 bpm (51–90 bpm). On average, over all healthy volunteers, the magnitude of HR correction for native myocardium ranged from 0.03 ± 0.05 msec (≤0.17 msec) using 2P‐5 to 12 ± 9 msec (≤30 msec) using 2P‐2, while the magnitude of HR correction for native blood ranged from 3 ± 2 msec (≤8 msec) using 2P‐5 to 14 ± 12 msec (≤41 msec) using 2P‐2.

**Figure 4:**
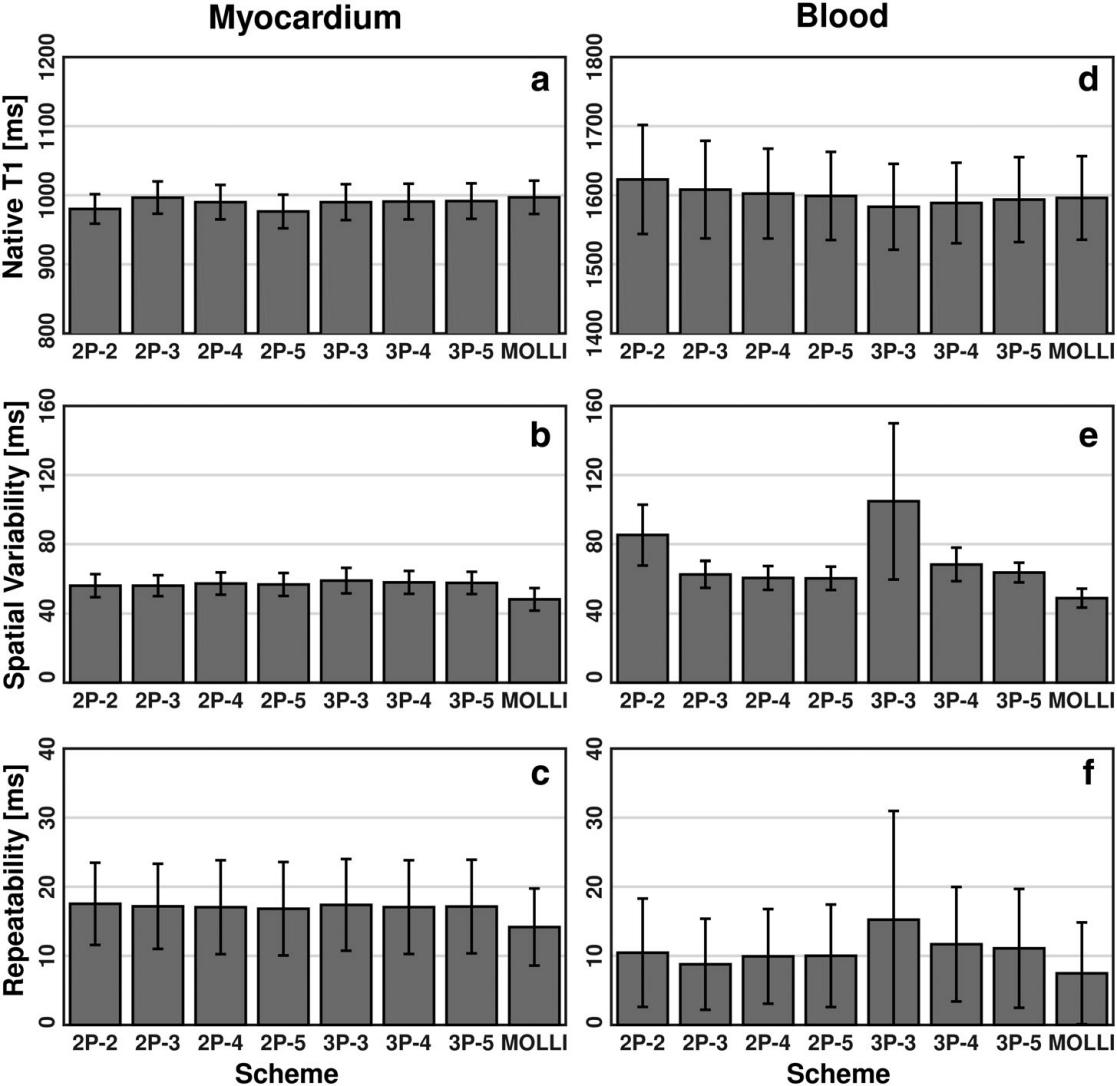
Native T_1_ times, spatial variability, and repeatability for myocardium (**a–c,** respectively) and blood (**d–f,** respectively) obtained using all T_1_ mapping schemes in 16 healthy volunteers. Average (bar plots) and SD (error bars) over all healthy volunteers are presented. There were no statistically significant differences between native myocardial and blood T_1_ times (*P* = 0.21 and *P* = 0.79, respectively) and repeatability (*P* = 0.87 and 0.41, respectively) obtained using all schemes. All shortened T_1_ mapping schemes led to increased myocardial and blood T_1_ spatial variability with respect to MOLLI (*P* < 0.0001).

Over all healthy volunteers, only one of 256 myocardial segments (0.4%) was discarded from the analysis. There were no statistically significant differences between all schemes in terms of native myocardial T_1_ times (*P* = 0.21), which were all in the range of 977–997 msec (Fig. 4a). There were no statistically significant differences between all shortened T_1_ mapping schemes in terms of myocardial T_1_ spatial variability (*P* = 0.87). However, they all had increased spatial variability by a factor of 1.2 with respect to MOLLI (56–59 msec vs. 48 msec, respectively, *P* < 0.0001) (Fig. 4b). There were no statistically significant differences between all schemes in terms of myocardial T_1_ repeatability, which were in the range of 14–18 msec (*P* = 0.87) (Fig. 4c).

Over all healthy volunteers, no statistically significant differences were found between all schemes in terms of native blood T_1_ times, which were in the range of 1583–1623 msec (*P* = 0.79) (Fig. 4d). 2P‐2 and 3P‐3 yielded higher spatial variability in the blood pool (85 msec and 105 msec, respectively) than the other shortened T_1_ mapping schemes (63 msec, *P* ≤ 0.0025), which were all inferior to MOLLI (49 msec, *P* < 0.0001) (Fig. 4e). There were no statistically significant differences between all schemes in terms of repeatability of native blood T_1_ times (*P* = 0.41), which were in the range of 7–15 msec for all schemes (Fig. 4f).

**Figure 5:**
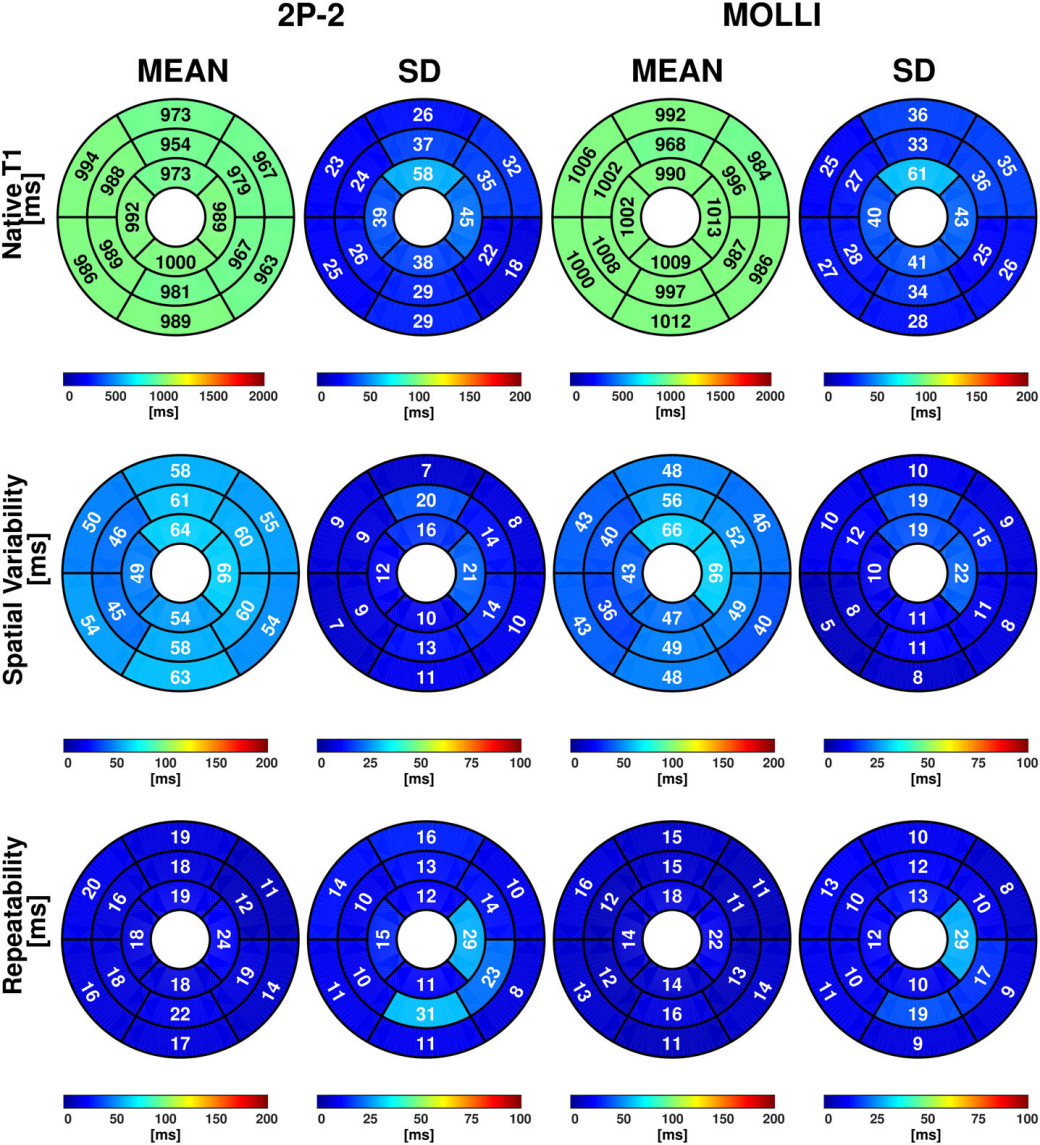
Segment‐wise native myocardial T_1_ times, spatial variability, and repeatability using 2P‐2 and MOLLI in 16 healthy volunteers. Data are shown as average ± SD over all healthy volunteers. No statistically significant differences were found between segmental values of native T_1_ times, spatial variability, and repeatability obtained using both methods.

Segment‐wise assessment of native myocardial T_1_ times, spatial variability, and repeatability of 2P‐2 and MOLLI are shown in Fig. 5. The segmental variation (SD over all myocardial segments) of native myocardial T_1_ times, spatial variability, and repeatability was of similar range between 2P‐2 and MOLLI [13/6/3 msec vs. 12/8/3 msec, respectively]).

### *Patient Study*

**Figure 6:**
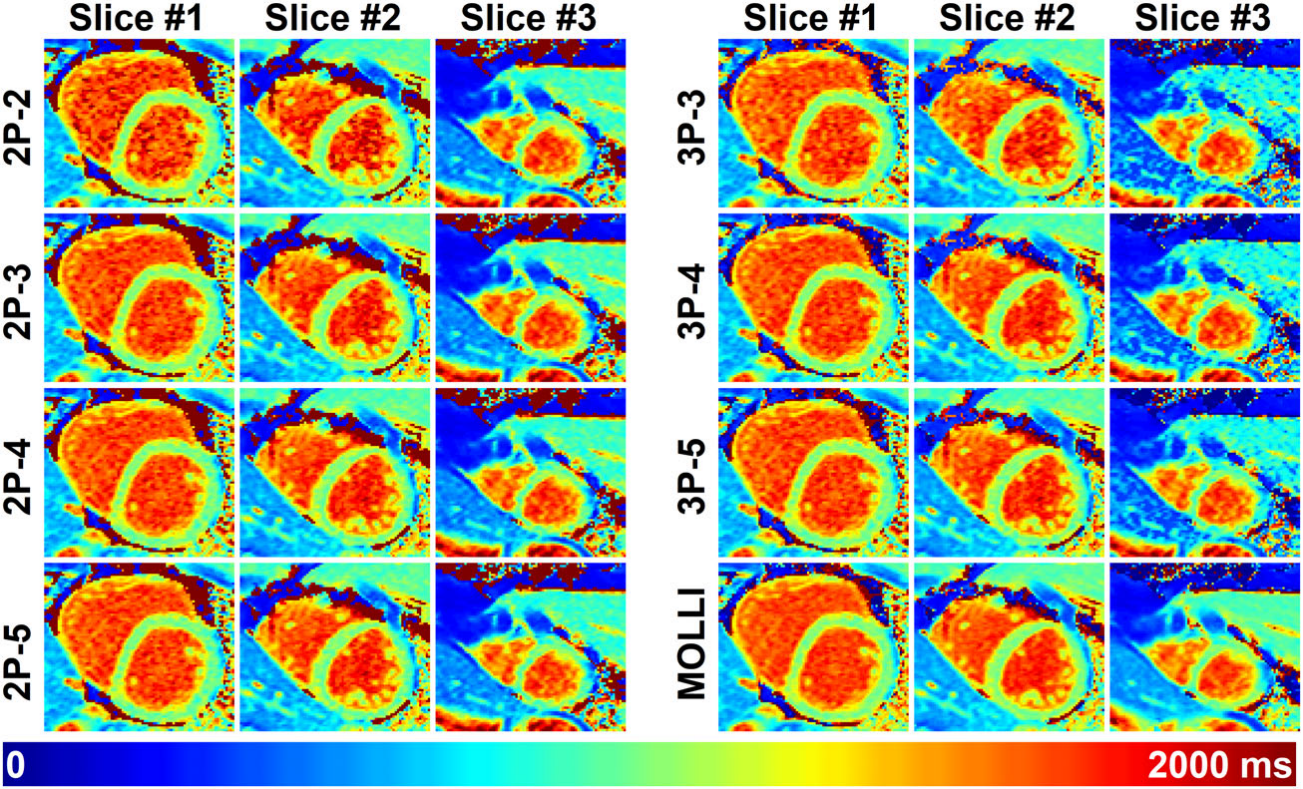
Example native myocardial T_1_ maps of a 38‐year‐old male patient (HR 84 bpm) admitted with syncope using all T_1_ mapping schemes. All schemes provided similar T_1_ map image quality and similar characteristics for native myocardial T_1_ times. Shortened schemes tended to have lower spatial homogeneity than MOLLI in the blood pool.

**Figure 7:**
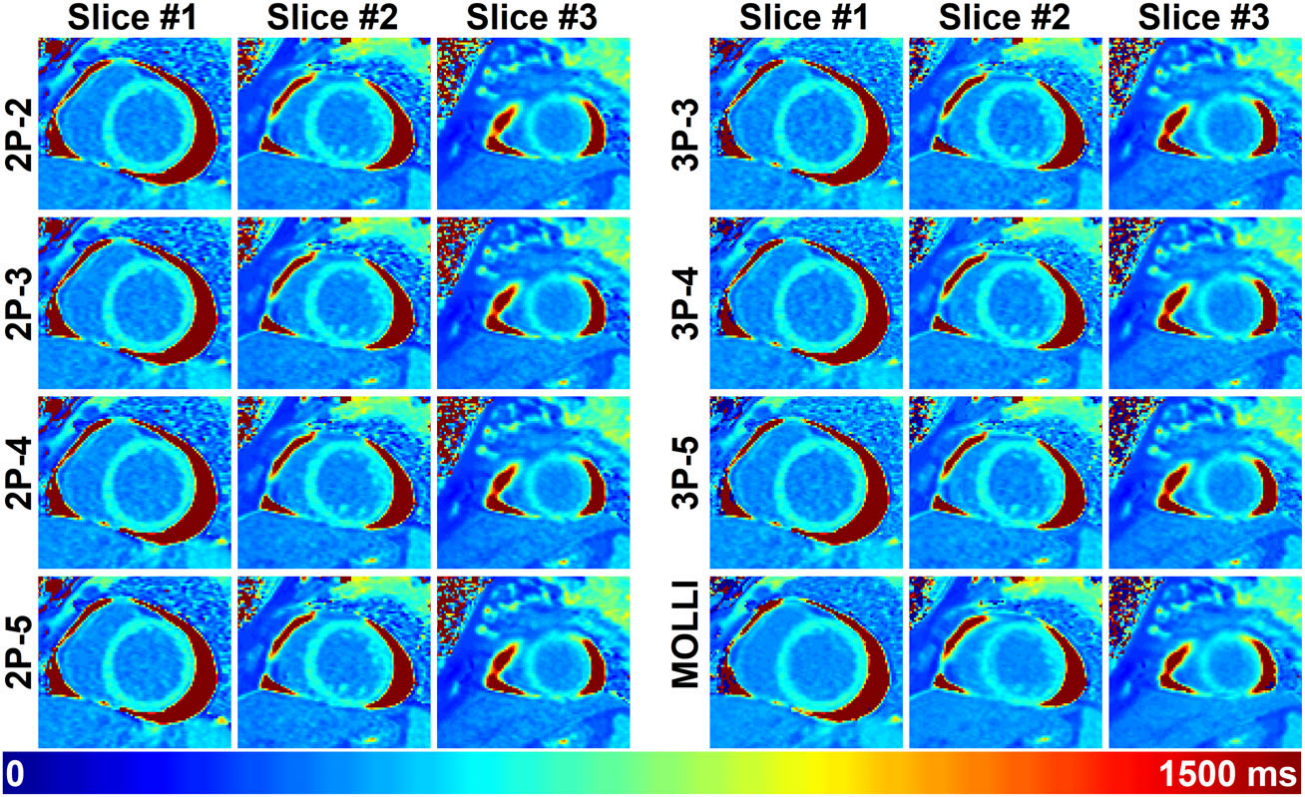
Example postcontrast myocardial T_1_ maps obtained in a 32‐year‐old female patient (HR 61 bpm) with severe left ventricular systolic dysfunction and pericardial effusion using all T_1_ mapping schemes. All schemes provided similar T_1_ map image quality as well as similar myocardial and blood T_1_ ranges across all slices.

Example native and postcontrast myocardial T_1_ maps obtained in two patients using all the evaluated T_1_ mapping schemes are shown in Figs. 6 and 7, respectively. Similar visual image quality and native myocardial T_1_ range were obtained for all schemes, although a higher perceived noise level can be observed in the left ventricular blood pool using shortened T_1_ mapping schemes.

Over all patients, the average HR was 68 ± 14 bpm (36–98 bpm). In all, 34 of 384 myocardial segments (9%) from five patients for native T_1_ mapping and 11 of 288 myocardial segments (4%) from two patients for postcontrast T_1_ mapping were discarded from the quantitative analysis due to substantial artifacts and/or motion. HR correction of native myocardial T_1_ times led to changes from 0.04 ± 0.06 msec (≤0.25 msec) using 2P‐5 to 13 ± 12 msec (≤43 msec) using 2P‐2, while HR correction of native blood T_1_ times led to changes from 3 ± 3 msec (≤11 msec) using 2P‐5 to 14 ± 13 msec (≤47 msec) using 2P‐2. The magnitude of HR correction for postcontrast myocardial and blood T_1_ times was <4 msec using all schemes.

**Figure 8:**
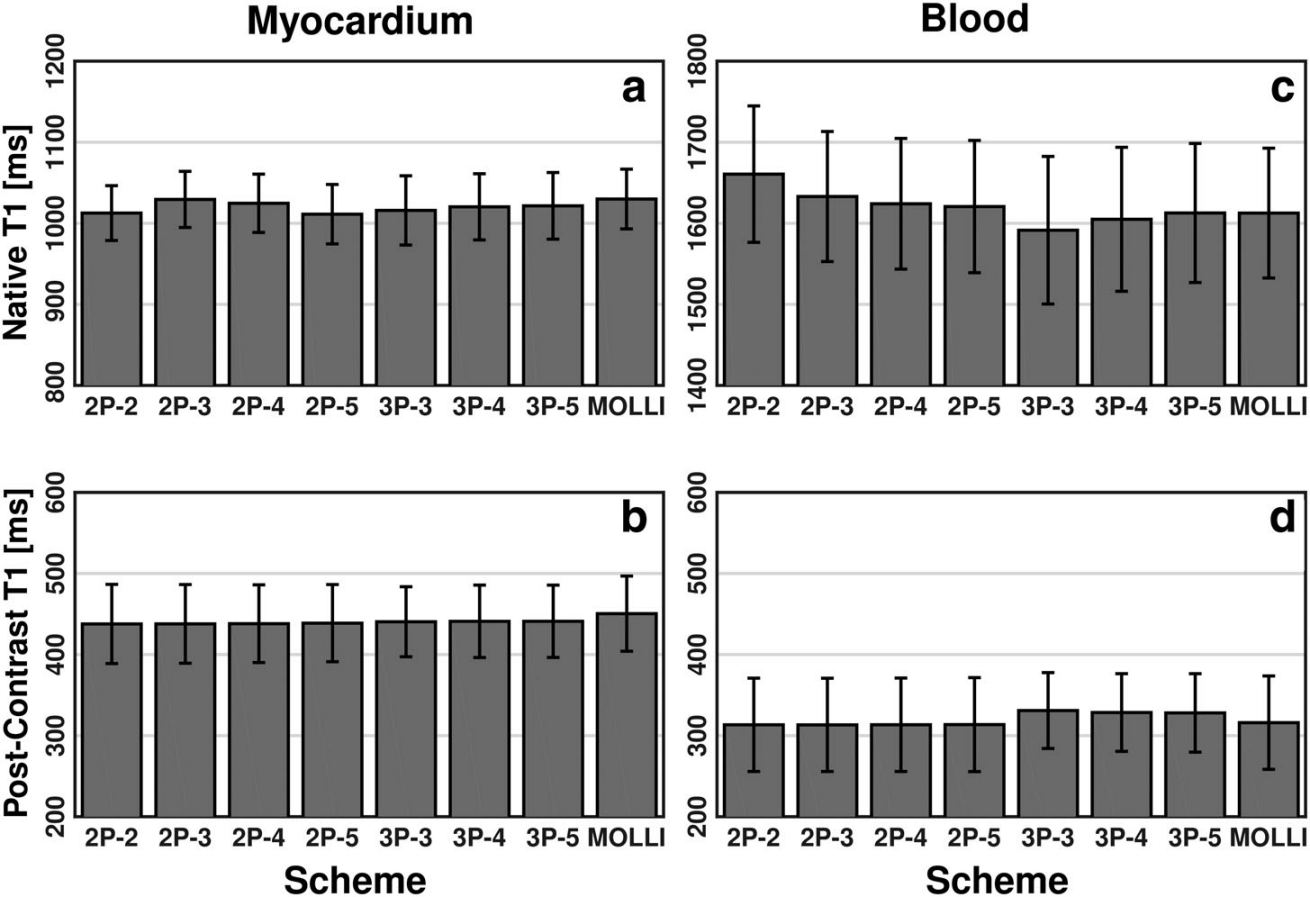
Native/postcontrast myocardial/blood T_1_ times (**a–d,** respectively) in 24 patients using all T_1_ mapping schemes. Average (bar plots) and SD (error bars) over all patients are presented. All methods led to similar range of native/postcontrast myocardial/blood T_1_ times (*P* ≥ 0.19).

Subject‐wise native and postcontrast T_1_ times for myocardium and blood using all schemes are shown in Fig. 8. There were no statistically significant differences between all schemes for each of these four T_1_ ranges (native/postcontrast myocardial/blood) (*P* ≥ 0.19).

**Figure 9:**
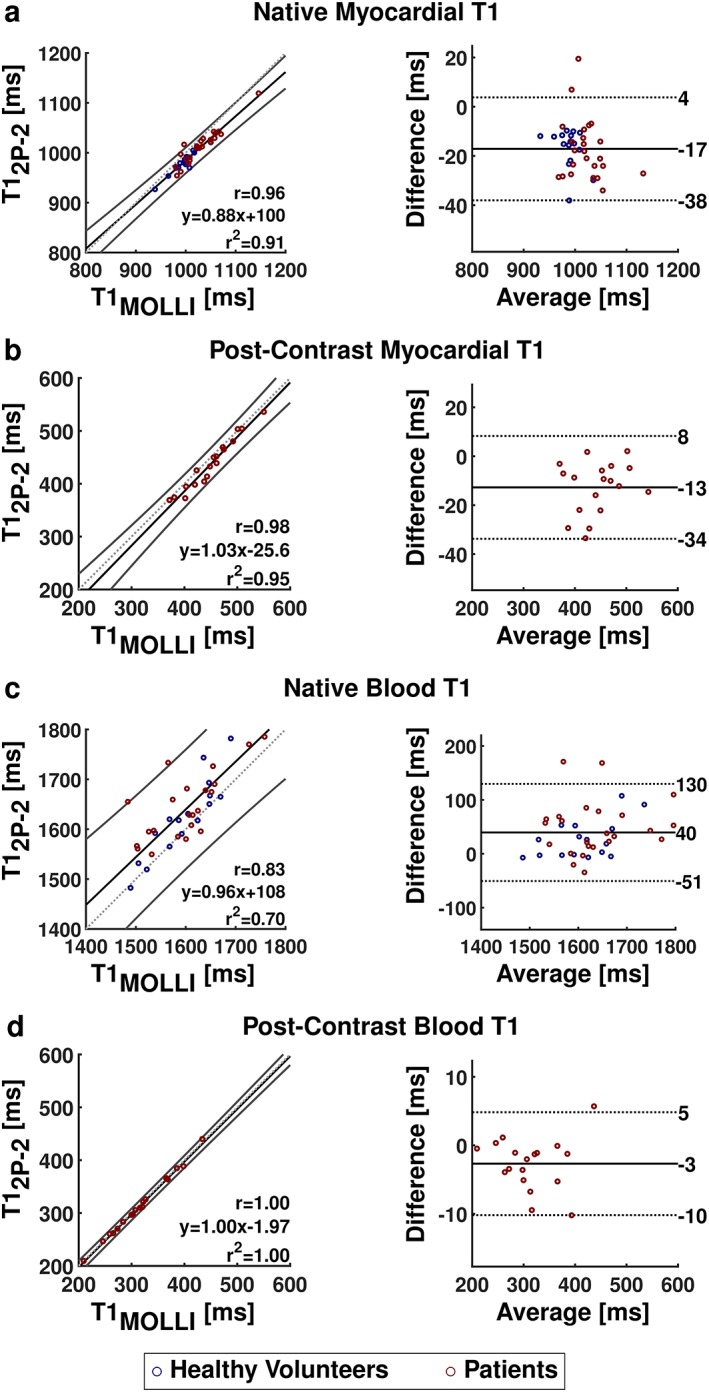
Correlation and agreement tests between T_1_ times obtained using 2P‐2 and MOLLI in all subjects including 16 healthy volunteers and 24 patients. Subfigure **(a–d)** illustrate Pearson correlation analysis and Bland–Altman plot of native myocardial T_1_ times, native blood T_1_ times, postcontrast myocardial T_1_ times, and postcontrast blood T_1_ times, respectively. Strong correlation and good agreement were found between T_1_ times obtained using 2P‐2 and MOLLI. In Pearson correlation analysis plots, confidence interval (solid lines) and identity line (y = x, dashed line) are also plotted besides the linear regression line (solid line). Correlation information including the Pearson correlation coefficient (r‐value), linear regression relationship (y as a function of x), and coefficient of determination (r^2^) is also displayed in Pearson correlation plots. In Bland–Altman plots, "average" stands for (T1_2P‐2_ + T1_MOLLI_)/2 and "difference" stands for (T1_2P‐2_‐T1_MOLLI_).

The Pearson correlation and Bland–Altman analyses in terms of subject‐wise native myocardial/blood T_1_ times (healthy volunteers and patients) and postcontrast myocardial/blood T_1_ times (patients only) are shown in Fig. 9 (only 2P‐2 vs. MOLLI) and Table 1 (each shortened scheme vs. MOLLI). Strong correlation was observed between 2P‐2 and MOLLI. The Pearson correlation coefficient between 2P‐2 and MOLLI for native blood T_1_ times was 0.83, and was ≥0.96 for all other T_1_ ranges. All other shortened T_1_ mapping schemes were also strongly correlated with MOLLI for each T_1_ range (Pearson correlation coefficient ≥ 0.90). For native blood T_1_ times, 2P‐2 and MOLLI were in moderate agreement (bias of 40 msec, 95% limits of agreement: –51 msec to 130 msec). For other T_1_ ranges, 2P‐2 and MOLLI were in good agreement with limited bias magnitude (≤17 msec) and narrow width of 95% limits of agreement (<43 msec). All other shortened T_1_ mapping schemes were in good agreement with MOLLI for native myocardial T_1_ mapping, with limited bias magnitude (≤19 msec) and narrow width of 95% limits of agreement (<39 msec).

**Table 1:** Pearson Correlation Analysis and Bland–Altman Plot Results

	Native myocardial T1	Native blood T1	Postcontrast myocardial T1	Postcontrast blood T1
2P‐2	0.96	0.83	0.98	1.00
	–17 ± 11 (–38,4)	40 ± 46 (–51,130)	–13 ± 11 (–34,8)	–3 ± 4 (–10,5)
2P‐3	0.97	0.94	0.97	1.00
	0 ± 8 (–17,16)	17 ± 26 (–33,67)	–13 ± 11 (–34,9)	–3 ± 4 (–10,4)
2P‐4	0.98	0.98	0.97	1.00
	–6 ± 7 (–20,8)	9 ± 15 (–21,39)	–12 ± 11 (–34,9)	–3 ± 4 (–10,5)
2P‐5	0.98	0.99	0.97	1.00
	–19 ± 7 (–33,–6)	6 ± 12 (–18,30)	–12 ± 12 (–34,11)	–2 ± 4 (–10,5)
3P‐3	0.97	0.96	0.97	0.99
	–11 ± 10 (–31,8)	–18 ± 23 (–62,26)	–10 ± 11 (–31,11)	15 ± 14 (–12,42)
3P‐4	0.98	0.99	0.98	0.99
	–8 ± 7 (–23,6)	–8 ± 13 (–33,18)	–9 ± 9 (–27,8)	12 ± 13 (–13,38)
3P‐5	0.98	1.00	0.98	0.98
	–7 ± 8 (–23,9)	–1 ± 8 (–16,15)	–9 ± 9 (–27,8)	12 ± 13 (–14,38)

Measured between each shortened T1 mapping scheme and MOLLI for native/postcontrast myocardial/blood T1 times. Data shown are as follows: first row, Pearson correlation coefficient as the *r*‐value; second row, bias ± SD (95% limits of agreement) in msec. All *P*‐values in Pearson correlation analysis are <0.0001.

Supplementary Material 8 shows native myocardial T_1_ maps obtained in a patient who was unable to sustain a stable breath‐hold for the entire duration of the acquisition. MOLLI led to substantial T_1_ map artifacts in both mid‐ventricular and apical slices, which were then discarded for all schemes from the quantitative analysis. All shortened T_1_ mapping schemes provided improved map quality in this patient.

## Discussion

In this work, we proposed and evaluated shortened T_1_ mapping schemes combined with a novel 2P fitting model for myocardial T_1_ mapping. These methods were successfully evaluated in numerical simulations, phantom, healthy volunteers, and patients. Compared with the conventional MOLLI 5‐(3)‐3 scheme, shortened T_1_ mapping schemes (down to two heartbeats only) combined with the proposed 2P fitting model resulted in no significant differences in terms of T_1_ estimates and repeatability and had similar T_1_ ranges as well as limited reduction of precision/increase of spatial variability. Importantly, the resulting native/postcontrast myocardial/blood T_1_ times measured by all shortened T_1_ mapping schemes were highly linearly correlated with the corresponding values measured using MOLLI. Finally, the proposed GPU implementation of the exhaustive search‐based optimization of the 2P fitting model enables fast T_1_ map reconstruction, which is suitable for clinical application.

In vivo myocardial T_1_ times, precision, and repeatability of MOLLI were in good agreement with previous works.^6^, ^14^, ^32^ All evaluated shortened T_1_ mapping schemes provided T_1_ times in a similar range as MOLLI for all native/postcontrast myocardial/blood T_1_ ranges. Moreover, all proposed shortened T_1_ mapping schemes have the same acquisition manner as 5‐(3)‐3 MOLLI. These observations suggest that, similar to MOLLI, these shortened T_1_ mapping schemes are also sensitive to T_2_ relaxation,^14^, ^18^ magnetization transfer,^19^ and off‐resonance effects.^16^ Furthermore, this work was performed at 1.5T. The potential of these shortened T_1_ mapping schemes at higher fields such as 3T remains to be demonstrated and will be the focus of future work.

The feasibility of the shortened T_1_ mapping schemes for postcontrast myocardial T_1_ mapping was demonstrated in numerical simulations, phantom, and patients. The spatial variability penalty of shortened T_1_ mapping schemes was more pronounced for typical short postcontrast T_1_ times than typical native myocardial T_1_ times, which could be interpreted as a consequence of lacking a second short‐TI image. For short T_1_ times (i.e., typical postcontrast T_1_ times), all 2P‐n schemes had higher precision and lower spatial variability than the shortened 3P‐n schemes. Therefore, 2P‐n schemes may be advantageous over shortened 3P‐n schemes in the context of ECV mapping.

The healthy volunteer study demonstrated that all proposed shortened T_1_ mapping schemes resulted in an increase of T_1_ spatial variability (by a factor of 1.2) for native myocardial T_1_ times when compared with MOLLI. This increase in spatial variability between 3P‐5 (approximated ShMOLLI for native T_1_) and MOLLI is in good agreement with previous comparison of ShMOLLI and MOLLI at 1.5T.^5^ Importantly, the use of fewer images for both 2P‐n and 3P‐n (i.e., *n* = 2/3/4) did not result in a further increase of spatial variability (i.e., precision loss) of native myocardial T_1_ time estimates when compared with 3P‐5. This suggests that the impact of each T_1_‐weighted image in the fitting process is dependent on its corresponding TI, similar to findings observed for saturation recovery‐based techniques.^33^, ^34^ Long TI images (i.e., with TI >> T_1_ range of interest) have reduced T_1_‐weighted contrast and may thus have reduced contributions to the precision of T_1_ estimates.

For long T_1_ times such as in vivo native blood T_1_ times, 2P‐2 led to a larger increase of spatial variability by a factor of 1.7 with respect to MOLLI, while other 2P‐n schemes still maintained a limited increase of T_1_ spatial variability by a factor < 1.28. This could be explained by the lack of sampling of long TI times in the 2P‐2 scheme. Therefore, 2P‐3 could be a valuable alternative to 2P‐2 for native T_1_ mapping, as it offers lower spatial variability for blood T_1_ quantification. However, blood T_1_ time is usually measured as the spatial average over a large ROI, which may mitigate this effect for 2P‐2, as no statistically significant differences were found in terms of repeatability for blood T_1_ quantification among all methods.

Myocardial T_1_ mapping based on the acquisition of only two images has been previously proposed using the AIR technique, which is based on saturation recovery.^9^ However, this technique was shown to considerably increase spatial variability of native myocardial T_1_ mapping by a factor of 2.5 when compared with MOLLI.^35^ The proposed 2P‐2 approach resulted in a limited increase of spatial variability for native myocardial T_1_ mapping by a factor of 1.2 when compared with MOLLI, and may thus be a valuable alternative for myocardial T_1_ mapping, as the acquisition can be performed in just two heartbeats.

The proposed 2P model‐based fitting technique enables correction for surface coil sensitivity variations using the normalization step of the dictionary matching. Exhaustive search‐based optimization over the entire range of physiological myocardial and blood native/postcontrast T_1_ times guarantees finding the global minimum of the cost function over a least‐square optimization, and was successfully used with the 2P model. Although such an approach is more computationally intensive, the proposed GPU‐based implementation substantially reduced the computation time to the subsecond scale per map, which is suitable for clinical application. Our results demonstrate that GPUs are particularly well suited for the reconstruction of T_1_ maps. This finding is in good agreement with prior studies where GPU‐based reconstruction substantially reduced the computation time of standard MOLLI reconstructions.^36^ Further reduction of the computation time may be achieved using GPU cards with higher performance or advanced dictionary search approaches such as fast group matching algorithms.^37^

Although 2P‐2 provides a 5‐fold acceleration with respect to MOLLI, the overall acceleration rate in multislice protocols may be reduced by the required rest‐periods between breath‐holds. However, the repetition of two‐heartbeat breath‐holds may enable the use of shortened recovery periods, improve patient comfort, and increase the probability of successful breath‐holds.

Bloch equations simulation was only performed on the employed inversion pulse to determine the inversion factor in the proposed 2P fitting model. However, Bloch equations simulation could also be used for the whole pulse sequence to generate the signal dictionary.^24^, ^25^, ^26^ Such an approach could be used to model the effect of the 2D readouts and provide improved accuracy of the T_1_ estimates, which will be the focus of future work.

In this work, the slice profile of the inversion pulses was approximated by one flip angle. Alternatively, the use of subslice‐based simulations may improve the accuracy of the slice profile correction.^25^ Estimation of the inversion factor could include a larger B_0_ range if fatty tissues are considered. The use of a weighted average over the B_0_ and B_1_ ranges could also be considered to further improve the accuracy of the inversion factor estimates.

In this work, PSIR was employed for all reconstructions. Alternatively, a multifitting approach could have been used for all these reconstructions.^2^ However, in our preliminary results (data not shown), we observed that the multifitting approach tended to fail to recover the correct signal polarity for the 3P fitting model in the presence of short T_1_ times. Although this technique was found robust for the 2P fitting model with all T_1_ ranges, we decided to use the PSIR approach for uniformity consideration.

No in‐plane motion correction was employed in this work. Image registration algorithms may provide different performance based on the amount and contrast of images used during the registration process. Therefore, to prevent such bias during in vivo evaluation of T_1_ spatial variability and repeatability, we decided to discard the registration step of the reconstruction and discard datasets with inappropriate breath‐hold. Nevertheless, retrospective image registration has been shown to improve the robustness of myocardial T_1_ mapping.^38^, ^39^ Therefore, the design of tailored image registration algorithms for the proposed shortened T_1_ mapping schemes will be the focus of future work. Finally, the use of shortened T_1_ mapping schemes has the potential to improve the native registration of the T_1_‐weighted images in patients unable to sustain long stable breath‐hold. This will be evaluated in future work in a larger patient cohort.

The evaluated shortened T_1_ mapping schemes showed varying degrees of HR dependence. These results are aligned with previous studies that demonstrated the HR dependence of MOLLI T_1_ times.^16^, ^29^, ^30^ The proposed HR correction models were found successful in reducing HR‐induced T_1_ variation to <10 msec for the entire T_1_ ranges. Alternative HR correction models have been proposed previously using a linear correction model based on measured T_1_ and HR.^29^, ^30^ In those studies, the slope and offset of the linear correction were assumed to be T_1_‐independent. Although we showed that a linear relationship between HR and measured T_1_ times is valid for a given T_1_ range, the linear regression slope and offset are also T_1_‐dependent. Therefore, T_1_‐dependent correction models were found more accurate than a simple T_1_‐independent linear model.

The HR dependence of T_1_ times is mainly due to the inaccuracies of the employed fitting models, partly caused by their T_2_ dependence.^16^ In this work, we found that the HR dependence of myocardial and blood T_1_ times were different, which could be explained by their large T_2_ difference (>150 msec vs. ~45 msec). Therefore, we decided to reconstruct two differently HR‐corrected T_1_ maps per slice: one with myocardium‐based HR correction and one with blood‐based HR correction. Automatic segmentation of the blood pool based on thresholding of T_1_ maps has been previously proposed for ECV quantification.^40^ Such an approach could allow the selection of the appropriate HR correction model on a per‐voxel basis and may be used to generate a single HR corrected T_1_ map for both the myocardium and blood.

This work has some limitations. First, the shortened T_1_ mapping schemes have been evaluated from a subset of a conventional 5‐(3)‐3 MOLLI scheme. This choice was made to minimize the number of required breath‐holds per subject. Second, the spatial variability measured as the SD over an ROI was used as a surrogate of the T_1_ precision, as commonly reported in prior studies.^5^, ^6^, ^14^, ^17^ However, this approach is susceptible to partial volume effects as well as artifacts, and thus may not fully represent the impact of noise in T_1_ time estimates. Third, trends were visually observed between MOLLI and the proposed shortened T_1_ mapping schemes in terms of spatial variability and repeatability in phantom. However, the differences did not reach statistical significance, which may be due to insufficient statistical power related to the limited number of vials available in our phantom. Fourth, the patient study was based on a small cohort of consecutive patients referred for clinical cardiac MRI. The benefit of these techniques in a larger patient cohort including patients with breath‐holding difficulties remains to be demonstrated and will be the focus of future studies. Furthermore, evaluation of this technique would be required in a cohort of patients with proven cardiac disease where mapping has clinical utility, such as with hypertrophic cardiomyopathy and Anderson‐Fabry's disease.

In conclusion, the proposed two‐heartbeat T_1_ mapping scheme yields a 5‐fold acceleration compared with MOLLI, with highly linearly correlated native/postcontrast myocardial/blood T_1_ times, no significant difference of repeatability, and a limited spatial variability penalty at 1.5T. This approach may be a valuable alternative for myocardial T_1_ mapping in patients with severe breath‐holding difficulties and reduce examination time of multislice protocols.

## Supporting information


**Supplementary Material 1** 5‐(3)‐3 MOLLI pulse sequence and PSIR T1 map reconstruction schemes. In the pulse sequence, ECG‐triggered 2D bSSFP multi‐TI imaging is performed for each slice. 2P‐n/3P‐n represent the T1 map reconstruction schemes using the proposed 2P/3P fitting models with the first n images in the acquisition order, respectively.
**Supplementary Material 2**. Simulated inversion recovery signal polarity as a function of T1 and T2 times for different flip angles (FAs). Bloch equations simulation of the sequence were used to determine the signal polarity of the shortest inversion time (TI) image (approximated as the signal polarity at the readout time of the k‐space center). Simulations were performed for different T1 times (range: 100‐300 ms), T2 times (range: 30‐300 ms) and FAs (range: 10‐85°). Red and blue regions indicate "positive" and “negative” polarities of the shortest TI image, respectively. Areas with negative polarity (blue) indicate conditions where the proposed PSIR assumption is valid. The minimum T1 times satisfying the proposed PSIR assumption increases with higher FAs and shorter T2 times. A T1 time of 172 ms was the lower bound ensuring the validity of the proposed PSIR assumption for the entire ranges of studied FAs/T2 times, which is smaller than the lower limit of the physiological ranges of native/post‐contrast T1 times in myocardium, blood and fat.
**Supplementary Material 3**. HR dependence of T1 estimates using 2P‐2 and MOLLI in phantom experiments. Each subfigure represents a different vial. The reference T1 and T2 values are given for each vial. Vial‐wise linear regression (dashed lines) of T1 vs. HR was performed for 2P‐2 (green) and MOLLI (black). Individual linear dependence of T1 on HR was observed for each vial. A stronger HR dependence was observed in the presence of long T1 times and short T2 times.
**Supplementary Material 4**. Dependence of T1‐HR linearity on T1 for 2P‐2 and MOLLI in phantom experiments. Parabolic regression (dashed curves) of slopes vs. offsets (cf. **Supplementary Material 3**) was performed for 2P‐2 (green) and MOLLI (black) on both short‐T2 and long‐T2 vials mimicking myocardium and blood, respectively.
**Supplementary Material 5**. Representative example of ROIs used for myocardial and blood T1 quantification. The blue contours represent the segmented myocardial region while the black contour represents the area used for blood T1 analysis.
**Supplementary Material 6**. Simulated T1 accuracy and precision as a function of T1 for different SNR. Although SNR had limited impact on T1 accuracy of all techniques, lower SNR resulted in precision penalty for all techniques as expected. However, SNR had limited influence on the relative precision penalty of all shortened T1 mapping schemes with respect to MOLLI which remained by a factor of 1.4‐1.5 for all SNR.
**Supplementary Material 7**. T1 variation over different HRs (40‐120 bpm) before and after the proposed HR‐correction using all T1 mapping schemes. Each subfigure represents a different vial. The reference T1 and T2 values are given for each vial. The proposed HR correction reduced T1 variations to a maximum of 7 ms in all cases.
**Supplementary Material 8**. Example native myocardial T1 maps obtained in a 32 yr female patient (HR 61 bpm) with severe left ventricular systolic dysfunction and pericardial effusion using all T1 mapping schemes (same patient as shown in Figure 7). This patient was unable to sustain a long stable breathhold for the entire duration of the acquisition of the mid‐ventricular and apical slices, which resulted in substantial artifacts in the corresponding MOLLI T1 maps (see black arrows). The shortened schemes provided good T1 map image quality for all slices.
