# Supplementary material for: Fast myocardial T1 mapping using shortened inversion recovery based schemes
Source: J Magn Reson Imaging. 2019 Jan 22;50(2):641–54. doi: 10.1002/jmri.26649 (PMC6751084; doi:10.1002/jmri.26649)
Supplement: Supplementary file 1 — Supplementary Material 1 5‐(3)‐3 MOLLI pulse sequence and PSIR T1 map reconstruction schemes. In the pulse sequence, ECG‐triggered 2D bSSFP multi‐TI imaging is performed for each slice. 2P‐n/3P‐n represent the T1 map reconstruction schemes using the proposed 2P/3P fitting models with the first n images in the acquisition order, respectively. Supplementary Material 2. Simulated inversion recovery signal polarity as a function of T1 and T2 times for different flip angles (FAs). Bloch equations simulation of the sequence were used to determine the signal polarity of the shortest inversion time (TI) image (approximated as the signal polarity at the readout time of the k‐space center). Simulations were performed for different T1 times (range: 100‐300 ms), T2 times (range: 30‐300 ms) and FAs (range: 10‐85°). Red and blue regions indicate "positive" and “negative” polarities of the shortest TI image, respectively. Areas with negative polarity (blue) indicate conditions where the proposed PSIR assumption is valid. The minimum T1 times satisfying the proposed PSIR assumption increases with higher FAs and shorter T2 times. A T1 time of 172 ms was the lower bound ensuring the validity of the proposed PSIR assumption for the entire ranges of studied FAs/T2 times, which is smaller than the lower limit of the physiological ranges of native/post‐contrast T1 times in myocardium, blood and fat. Supplementary Material 3. HR dependence of T1 estimates using 2P‐2 and MOLLI in phantom experiments. Each subfigure represents a different vial. The reference T1 and T2 values are given for each vial. Vial‐wise linear regression (dashed lines) of T1 vs. HR was performed for 2P‐2 (green) and MOLLI (black). Individual linear dependence of T1 on HR was observed for each vial. A stronger HR dependence was observed in the presence of long T1 times and short T2 times. Supplementary Material 4. Dependence of T1‐HR linearity on T1 for 2P‐2 and MOLLI in phantom experiments. Parabolic regression (dashe [file JMRI-50-641-s001.docx]

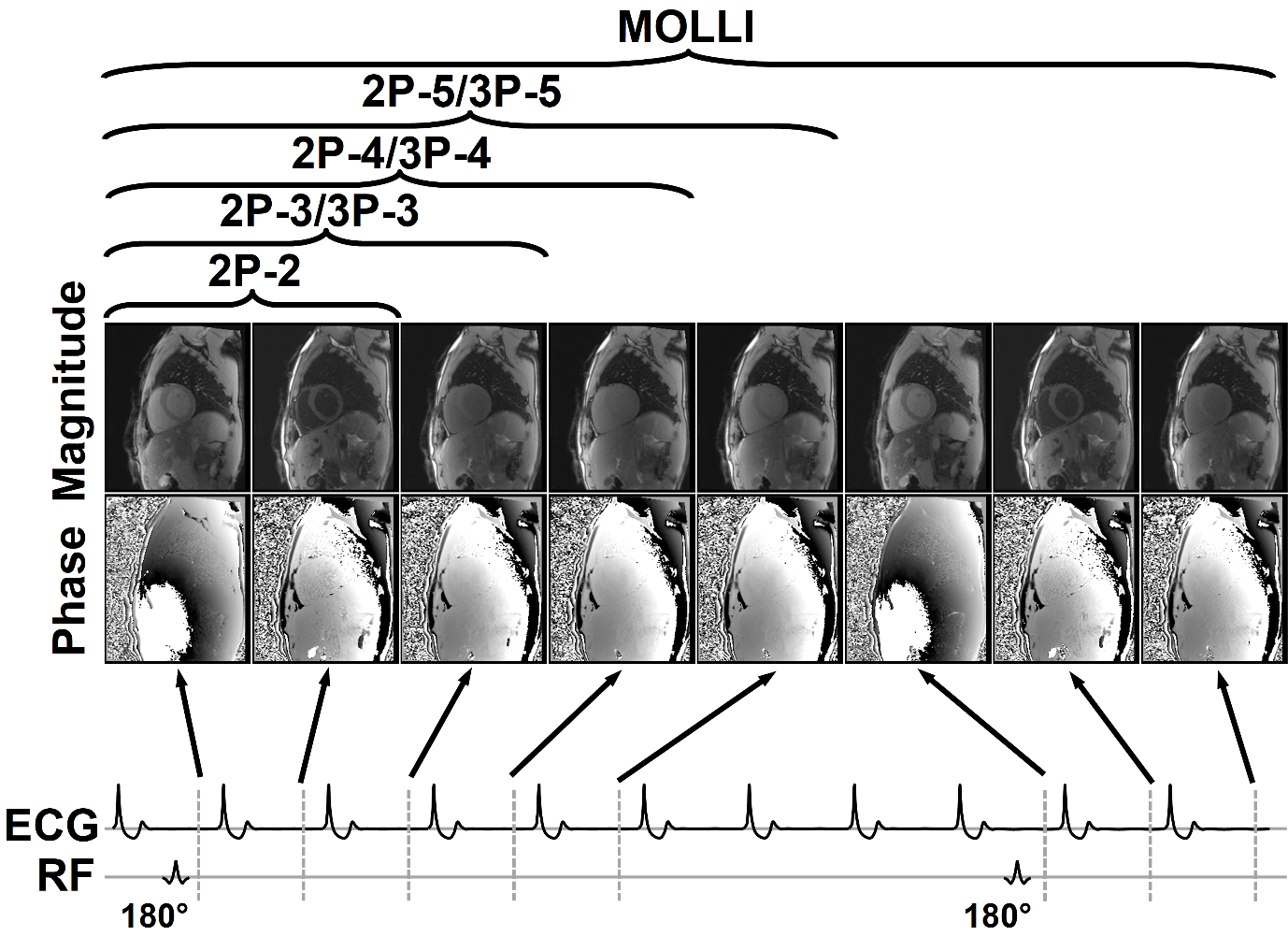


**Supplementary Material 1**. 5-(3)-3 MOLLI pulse sequence and PSIR T1 map reconstruction schemes. In the pulse sequence, ECG-triggered 2D bSSFP multi-TI imaging is performed for each slice. 2P-n/3P-n represent the T1 map reconstruction schemes using the proposed 2P/3P fitting models with the first n images in the acquisition order, respectively.


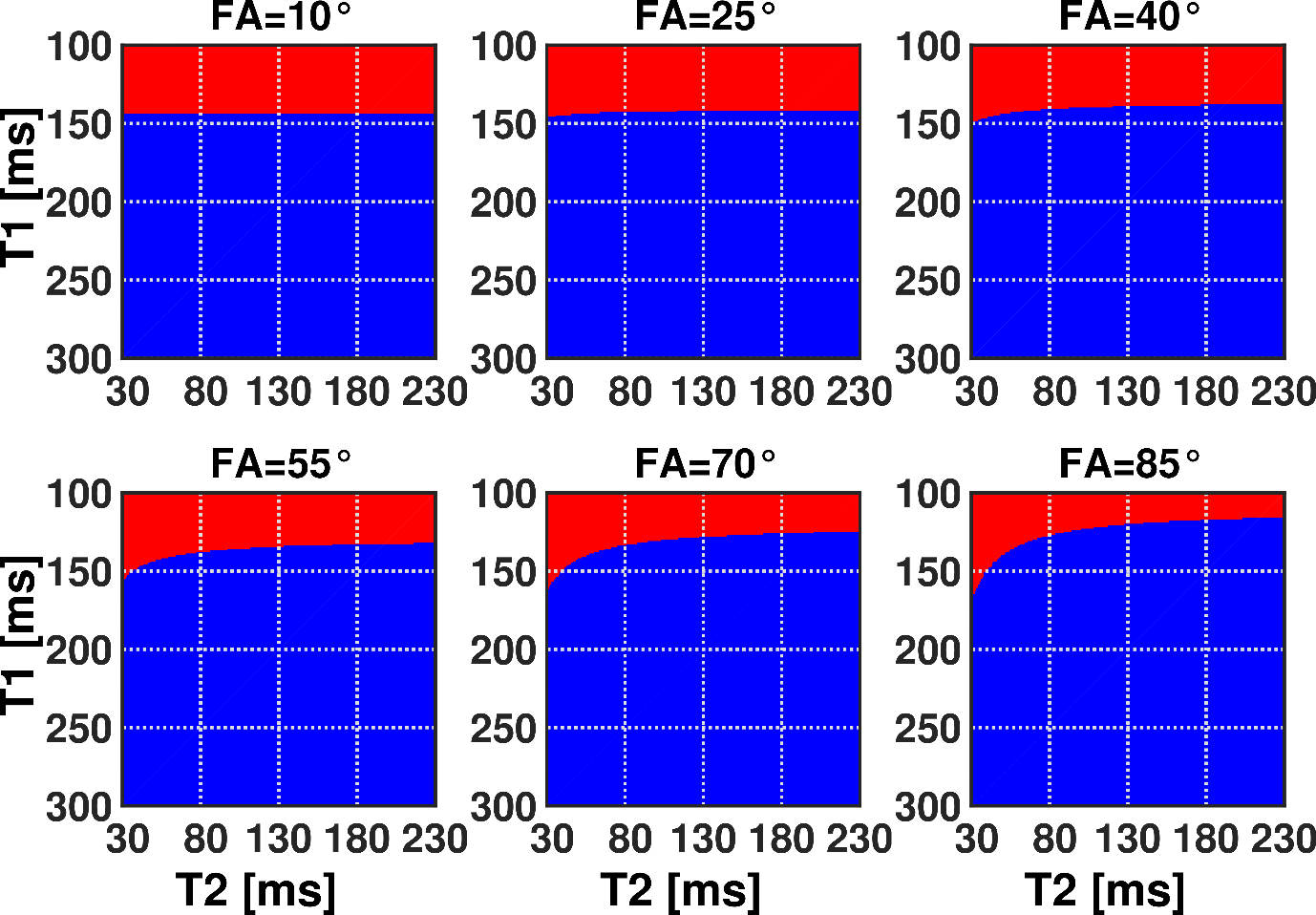


**Supplementary Material 2**. Simulated inversion recovery signal polarity as a function of T1 and T2 times for different flip angles (FAs). Bloch equations simulation of the sequence were used to determine the signal polarity of the shortest inversion time (TI) image (approximated as the signal polarity at the readout time of the k-space center). Simulations were performed for different T1 times (range: 100-300ms), T2 times (range: 30-300ms) and FAs (range: 10-85°). Red and blue regions indicate "positive" and “negative” polarities of the shortest TI image, respectively. Areas with negative polarity (blue) indicate conditions where the proposed PSIR assumption is valid. The minimum T1 times satisfying the proposed PSIR assumption increases with higher FAs and shorter T2 times. A T1 time of 172ms was the lower bound ensuring the validity of the proposed PSIR assumption for the entire ranges of studied FAs/T2 times, which is smaller than the lower limit of the physiological ranges of native/post-contrast T1 times in myocardium, blood and fat.


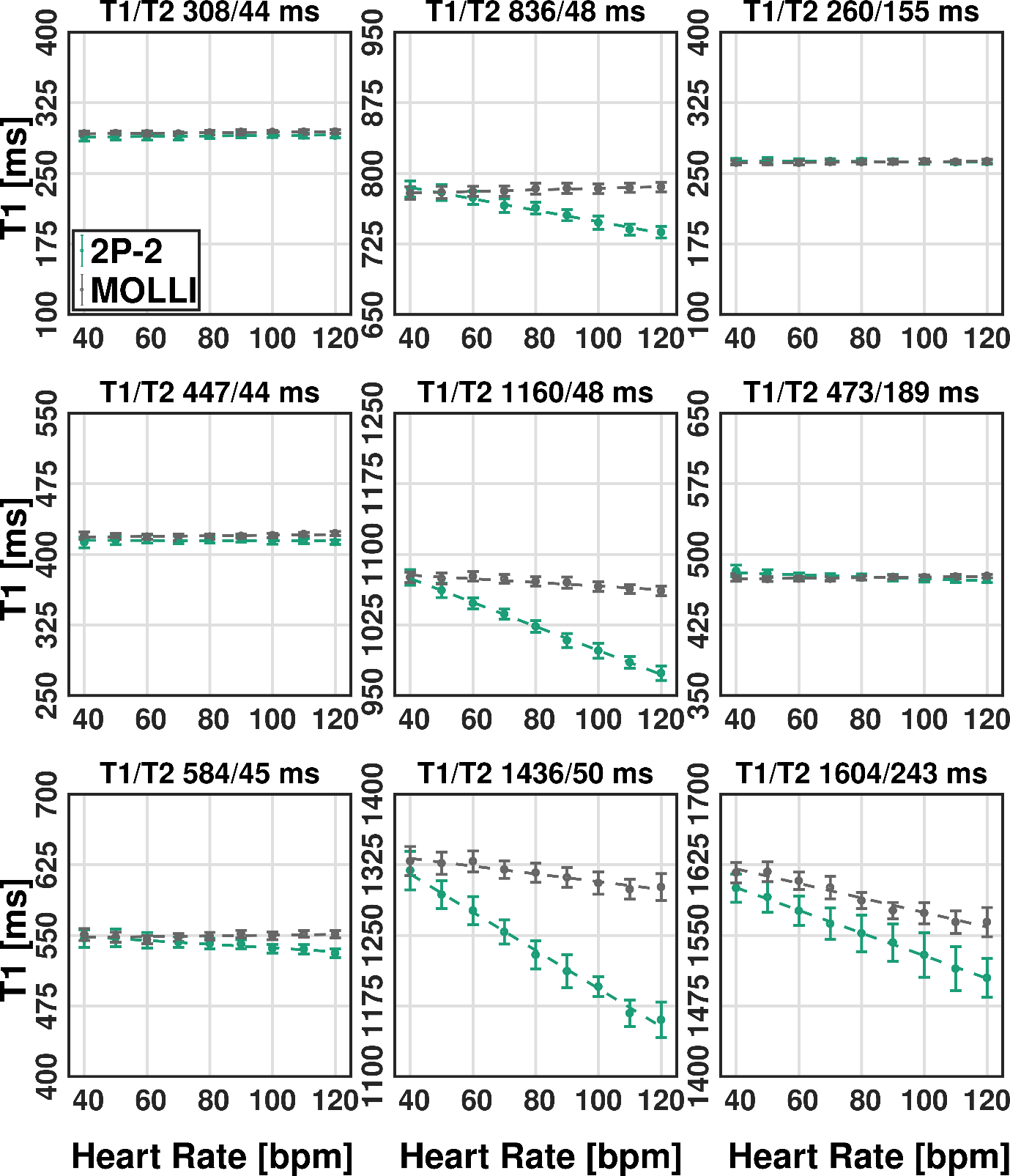


**Supplementary Material 3**. HR dependence of T1 estimates using 2P-2 and MOLLI in phantom experiments. Each subfigure represents a different vial. The reference T1 and T2 values are given for each vial. Vial-wise linear regression (dashed lines) of T1 vs. HR was performed for 2P-2 (green) and MOLLI (black). Individual linear dependence of T1 on HR was observed for each vial. A stronger HR dependence was observed in the presence of long T1 times and short T2 times.


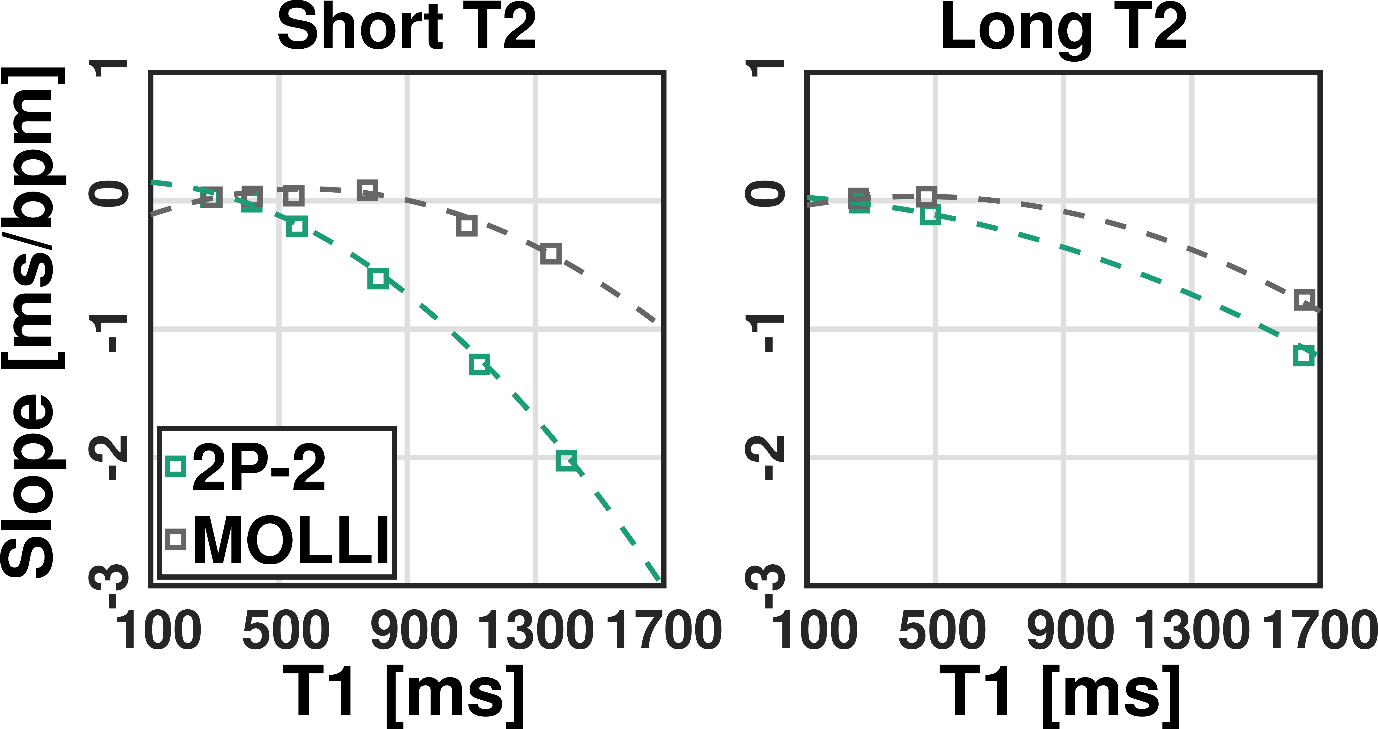


**Supplementary Material 4**. Dependence of T1-HR linearity on T1 for 2P-2 and MOLLI in phantom experiments. Parabolic regression (dashed curves) of slopes vs. offsets (cf. **Supplementary Material 3**) was performed for 2P-2 (green) and MOLLI (black) on both short-T2 and long-T2 vials mimicking myocardium and blood, respectively.


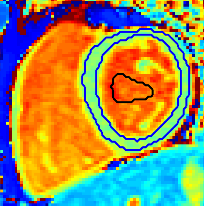


**Supplementary Material 5**. Representative example of ROIs used for myocardial and blood T1 quantification. The blue contours represent the segmented myocardial region while the black contour represents the area used for blood T1 analysis.


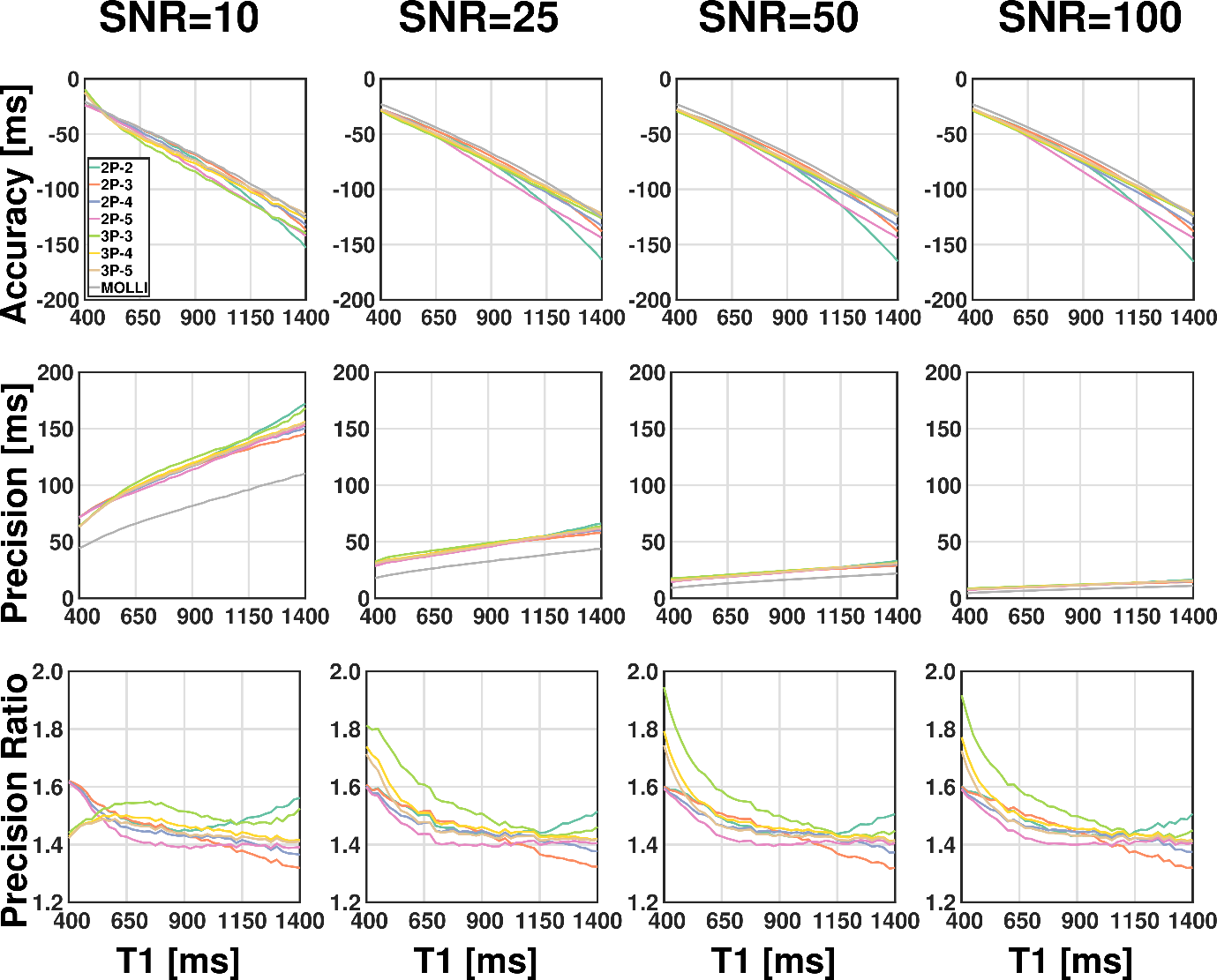


**Supplementary Material 6**. Simulated T1 accuracy and precision as a function of T1 for different SNR. Although SNR had limited impact on T1 accuracy of all techniques, lower SNR resulted in precision penalty for all techniques as expected. However, SNR had limited influence on the relative precision penalty of all shortened T1 mapping schemes with respect to MOLLI which remained by a factor of 1.4-1.5 for all SNR.


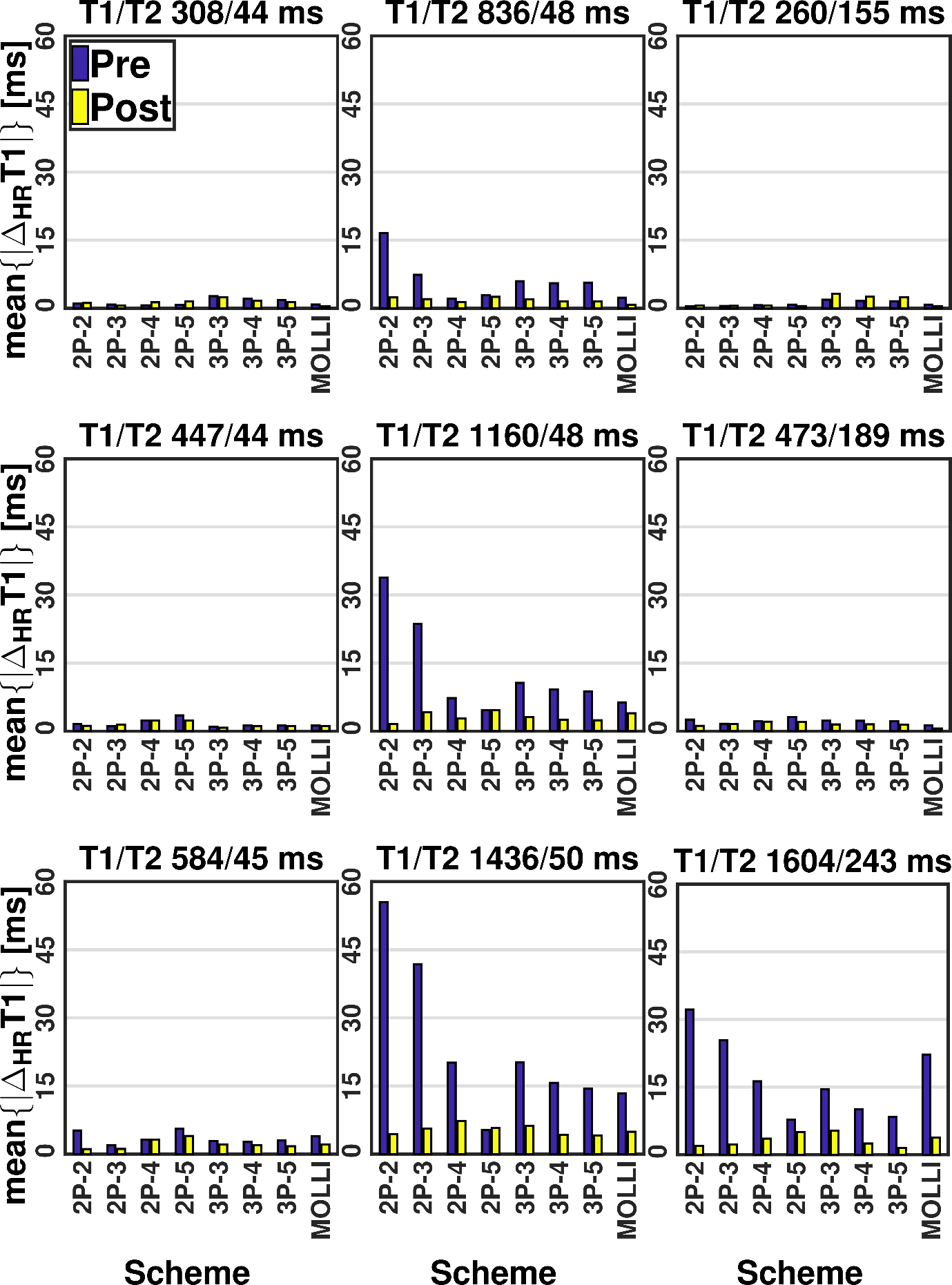


**Supplementary Material 7**. T1 variation over different HRs (40-120bpm) before and after the proposed HR-correction using all T1 mapping schemes. Each subfigure represents a different vial. The reference T1 and T2 values are given for each vial. The proposed HR correction reduced T1 variations to a maximum of 7ms in all cases.


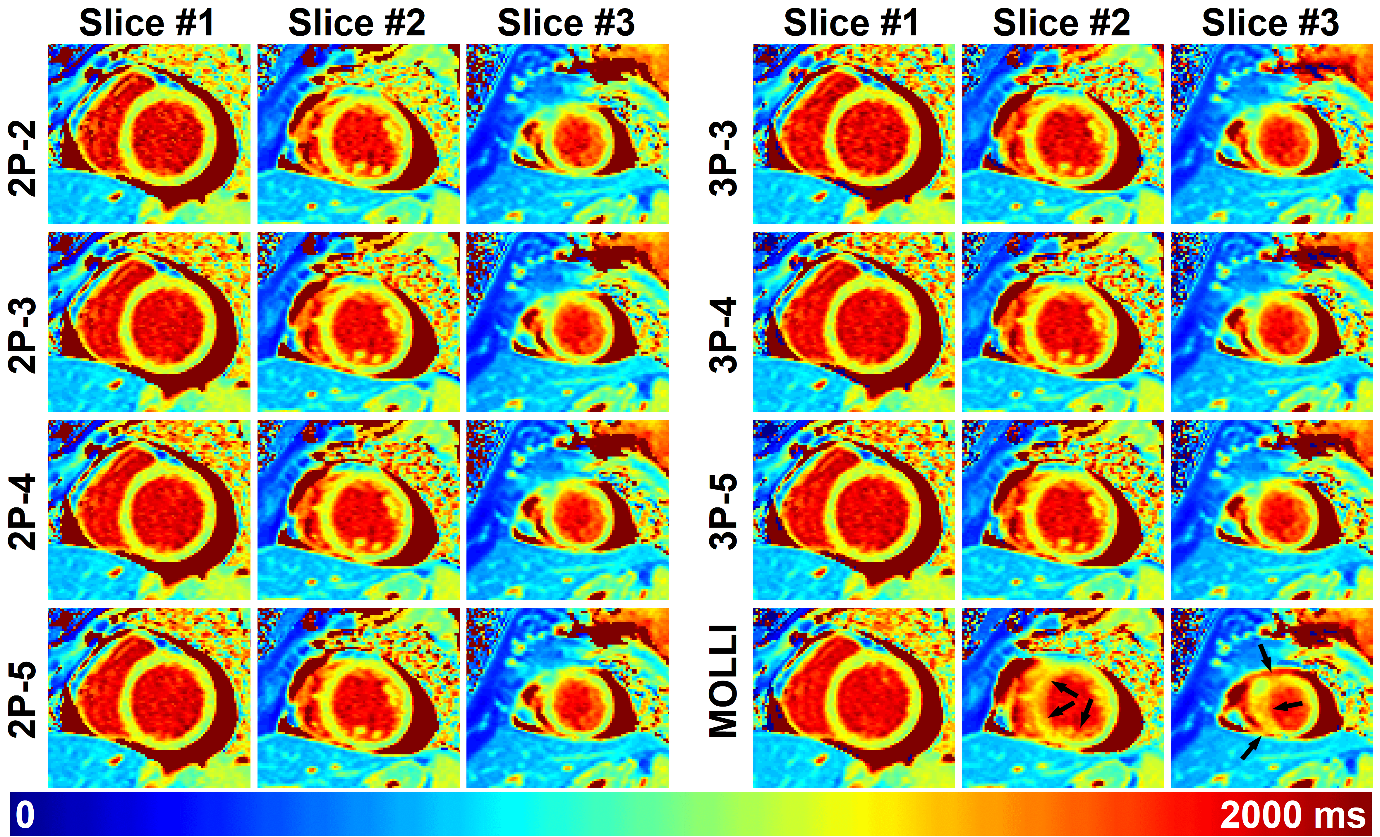


**Supplementary Material 8**. Example native myocardial T1 maps obtained in a 32yr female patient (HR 61bpm) with severe left ventricular systolic dysfunction and pericardial effusion using all T1 mapping schemes (same patient as shown in **Figure 7**). This patient was unable to sustain a long stable breathhold for the entire duration of the acquisition of the mid-ventricular and apical slices, which resulted in substantial artifacts in the corresponding MOLLI T1 maps (see black arrows). The shortened schemes provided good T1 map image quality for all slices.
